# Phylogenomic and morphological relationships among the botryllid ascidians (Subphylum Tunicata, Class Ascidiacea, Family Styelidae)

**DOI:** 10.1038/s41598-021-87255-2

**Published:** 2021-04-16

**Authors:** Marie L. Nydam, Alan R. Lemmon, Jesse R. Cherry, Michelle L. Kortyna, Darragh L. Clancy, Cecilia Hernandez, C. Sarah Cohen

**Affiliations:** 1 Math and Science Program, Soka University of America, 1 University Drive, Aliso Viejo, CA 92656 USA; 2 Department of Scientific Computing, Florida State University, 400 Dirac Science Library, Tallahassee, FL 32306 USA; 3 Department of Biological Science, Florida State University, 319 Stadium Drive, Tallahassee, FL 32306 USA; 4 Biology Department and Estuarine and Ocean Science Center, San Francisco State University, 3150 Paradise Drive, Tiburon, CA 94920 USA

**Keywords:** Phylogenetics, Evolution, Zoology

## Abstract

Ascidians (Phylum Chordata, Class Ascidiacea) are a large group of invertebrates which occupy a central role in the ecology of marine benthic communities. Many ascidian species have become successfully introduced around the world via anthropogenic vectors. The botryllid ascidians (Order Stolidobranchia, Family Styelidae) are a group of 53 colonial species, several of which are widespread throughout temperate or tropical and subtropical waters. However, the systematics and biology of this group of ascidians is not well-understood. To provide a systematic framework for this group, we have constructed a well-resolved phylogenomic tree using 200 novel loci and 55 specimens. A Principal Components Analysis of all species described in the literature using 31 taxonomic characteristics revealed that some species occupy a unique morphological space and can be easily identified using characteristics of adult colonies. For other species, additional information such as larval or life history characteristics may be required for taxonomic discrimination. Molecular barcodes are critical for guiding the delineation of morphologically similar species in this group.

## Introduction

Ascidians (Phylum Chordata, Class Ascidiacea) are a large group of invertebrates which occupy a central role in the ecology of marine benthic communities^1^. Many ascidian species have become successfully introduced around the world via anthropogenic vectors, with a few species causing extensive ecological and economic damage^2^. Invasive ascidians, including botryllids, have effected declines in native species richness^3^, altered benthic community structure^3–6^, and disrupted the link between pelagic and benthic communities^7,8^. Economically, ascidians are a major problem for the aquaculture industry: the species *Styela clava* was estimated to cost the New Zealand green-lipped mussel industry NZ$23.9 million over a 24-year period^9^.

The botryllid ascidians (Class Ascidiacea, Order Stolidobranchia, Family Styelidae) are a group of colonial species, of which 53 are currently described. *Botryllus schlosseri,* a model organism for allorecognition, regeneration, development, and genomics, has been spreading anthropogenically throughout temperate waters for at least 100 years^10,11^. This species thrives in artificial habitats such as aquaculture facilities and harbors^12–15^, but has also colonized natural habitats and therefore has the potential to affect rocky bottom and seagrass ecosystems^16,17^. Several other botryllid species have been spreading more recently. *Botrylloides violaceus* has been spreading from East Asia in temperate oceans in the last 50 years^18–21^. *Botrylloides niger* has low nucleotide diversity at a mitochondrial and a nuclear gene, indicating that its spread across the tropics may be similarly recent^22^. *Botrylloides diegensis* has been in southern California for at least 100 years^23^, but is thought to be native to the western or southern Pacific^18^ and is a recent invader to northwest Europe^24^. Finally, *Botrylloides giganteus* is widespread in the tropics but was only correctly identified in 2019^25^*.* All of these recent non-native species are spreading rapidly, and are able to tolerate a wide range of temperature and salinity regimes^19,22,25–29^. These species are highly likely to continue spreading, and will continue to shape the ecologies of the communities they invade.

Despite the global mobility of several botryllid species and the existence of *Botryllus schlosseri* as a model system, the overall systematics and biology of this group of ascidians are not well resolved. Botryllids are difficult to identify taxonomically because many of the species have very similar characteristics^30,31^, and morphologically-based species identification remains a challenge despite considerable effort^11,25,32–36^. More recently, combined molecular and morphological analyses have revealed the existence of new species and confirmed the taxonomic validity of already described species^24,25,37,38^. Moreover, DNA barcoding has allowed the identification of a new clade currently described as the “radiata morph” of *Botrylloides leachii,* while the clade previously reported in the nucleotide databases as *Botrylloides leachii* has been recognized as *Botrylloides diegensis*^24^.

The phylogenetic relationships among these species have not been studied since 2001^39,40^. Several of the relationships in the Cohen et al.^39^ eight taxon phylogeny were not well-supported because a single relatively low variation locus was used (18S rDNA), although the morphological distinction between the two genera, *Botryllus* and *Botrylloides* was supported. In the phylogeny presented in Ref.^40^ based on 18 s rDNA and four morphological characters, *Botrylloides* and *Botryllus* were also considered distinct, although this phylogeny includes mainly Japanese species and lacks support values (e.g. Bremer, jackknife or bootstrap support). Relationships involving the *Botryllus schlosseri* species complex have been investigated^40–42^. For instance, using mitochondrial cytochrome oxidase I (mtCOI), *Botrylloides niger* was determined to be an outgroup to *Botryllus schlosseri* Clades A–E, and *Botrylloides leachii* was an outgroup to the *Botrylloides niger/Botryllus schlosseri* clade^41^.

To provide a framework for future studies of both native and non-native botryllid ascidians and to examine the relationships among geographically restricted and widespread species, we have developed 200 nuclear markers and constructed a well-resolved phylogenomic tree using these loci. Probes corresponding to the 200 nuclear markers were hybridized to genomic libraries from 55 specimens, including previously undescribed species from the Caribbean Sea and the Philippines.

## Results

The results are presented in two parts: (1) phylogenetic relationships among botryllid species, and (2) morphological analyses in the botryllid group. These two sets of results were generated using distinct datasets. The phylogenomic tree was constructed using sequencing reads for 200 loci from 55 specimens of botryllids. Collection information and a unique identifier for each of the 55 specimens can be found in Table 1. The morphological dataset includes all 53 described botryllid species, plus four additional taxa that are not currently described in the literature (see Supplementary Fig. S1 for morphological descriptions of these four taxa). We compiled data on 31 morphological characters for each of these 57 species (see Supplementary Fig. S2), and used these characters in a Principal Components Analysis.

**Table 1:** Collection information for the 55 samples included in phylogenomic tree.

Specimen ID	Species	Collection location	Collection coordinates	Collection habitat	mtCOI Barcode Accession Number	Unique barcode	% identity of the next closest species	Date collected	Collector
Sb1	*Symplegma brakenhielmi*	Bocas del Toro, Panama	9° 24′ 52.8″ N 81° 49′ 12.3″ W	Mangrove roots	NA			01-07-2017	Marie Nydam
Sb2	*Symplegma brakenhielmi*	Bocas del Toro, Panama	9° 24′ 52.8″ N 81° 49′ 12.3″ W	Mangrove roots	MT232734	Yes	97%, *Symplegma* sp.	01-07-2017	Marie Nydam
Bp1	*Botryllus* sp.	Shizugawa, Japan	38° 38′ 41.2″ N 141° 28′ 34.6″ E	Intertidal	MW817931	Yes	93%, B. primigenus OTU 1S	07-07-2009	Erin Grey
Bsp1	*Botryllus* sp.	Maricaban Island (Bethlehem), Philippines	13° 40′ 18.6″ N 120° 50′ 43.6″ E	Coral Reef, on worm tube	MW817932	Yes	88%, Bsp2 from this study	22-03-2008	Beth Moore
Bsp2	*Botryllus* sp.	Heron Island, Australia	23° 26′ 31.5″ S 151° 54′ 53.4″ E	Intertidal	MT232726	Yes	88%, Bsp1 from this study	01-12-2011	Tony De Tomaso
Bsp3	*Botryllus* sp.	Fort Pierce (Little Jim Bridge), Florida, USA	27° 27′ 00.2″ N 80° 19′ 17.3″ W	Oyster Aquaculture Rack	NA			09-06-2011	Linda Walters
Bh1	*Botryllus horridus*	Miura, Japan	35° 09′ 38.0″ N 139° 36′ 46.2″ E	Docks	MT232732	Yes	84%, *B. conchyliatus*	16-07-2009	Erin Grey
Bga1	*Botryllus gaiae*	Falmouth, UK	50° 09′ 48.0″ N 5° 04′ 57.8″ W	Docks	KX500896	No	90%, *B. schlosseri* Clade D	30-05-2015	Marie Nydam
Bga2	*Botryllus gaiae*	Poole, UK	50° 42′ 47.8″ N 1° 57′ 22.8″ W	Docks	KX500856	No	90%, *B. schlosseri* Clade D	30-05-2015	Marie Nydam
Bsc1	Botryllus schlosseri	Ravenna, Italy	44° 29′ 21.2″ N 12° 17′ 13.4″ E	Docks	MW817933	Yes	86%, *B. gaiae*	27-05-2004	Joelle Tirindelli
Bsp5	*Botryllus* sp.	Bocas del Drago, Panama	9° 24′ 51.8″ N 82° 19′ 51.1″ W	Coral Reef	MW8179 34	Yes	88%, *B. *cf. *anceps*	01-07-2017	Rosana Rocha
Bv1	*Botrylloides violaceus*	Usujiri, Japan	41° 56′ 56.9″ N 140° 56′ 08.1″ E	Intertidal	NA			21-07-2009	Erin Grey
Bv2	*Botrylloides violaceus*	Asamushi, Japan	40° 53′ 25.9″ N 140° 51′ 29.9″ E	Docks	NA			07-07-2009	Erin Grey
Bv3	*Botrylloides violaceus*	Shizugawa, Japan	38° 38′ 41.2″ N 141° 28′ 34.6″ E	Intertidal	NA			25-07-2009	Erin Grey
Bv4	*Botrylloides violaceus*	Drakes Estero, CA, USA	38° 03′ 20.6″ N 122° 56′ 28.1″ W	Oyster Aquaculture Rack	NA			Unknown	SERC
Bv5	*Botrylloides violaceus*	Alameda, CA, USA	37° 45′ 59.1″ N 122° 16′ 28.0″ W	Seagrass beds (*Zostera marina)*	NA			08-15-2003	Sarah Cohen, Esa Crumb
Bv6	*Botrylloides violaceus*	Tiburon, CA, USA	37° 53′ 21.6″ N 122° 26′ 50.2″ W	Dock-Paradise Cay	MW8179 30	Yes	81%, *B. schlosseri*	06-02-2004	Richard Coleman, Gail Ashton, and Verena Wang
Bgi1	*Botrylloides giganteus*	Peru	Exact location unknown	Pilings	NA			2010 and 2011	Vanessa Guerra
Bgi2	*Botrylloides giganteus*	Chula Vista, CA, USA	32° 37′ 34.4″ N 117° 06′ 08.7″ W	Docks	MT232725	No	83%, Botrylloides diegensis	18–06-2008	Gail Ashton, Sarah Cohen, Verena Wang
Bgi3	*Botrylloides giganteus*	San Diego Bay, CA, USA	32° 42′ 34.8″ N 117° 13′ 59.3″ W	Docks	NA			16-06-2008	Verena Wang, Sarah Cohen, Gail Ashton
Bf1	*Botrylloides fuscus*	Miura, Japan	35° 09′ 38.0″ N 139° 36′ 46.2″ E	Docks	MT232731	Yes	85%, *B. perspicuus*	16-07-2009	Erin Grey
Bf2	*Botrylloides fuscus*	Ebisu-jima, Japan	34° 39′ 09.2″ N 138° 57′ 53.9″ E	Intertidal	MW817936	Yes	85%, *B. jacksonianum*	25-06-2009	Erin Grey
Bf3	*Botrylloides fuscus*	Ebisu-jima, Japan	34° 39′ 09.2″ N 138° 57′ 53.9″ E	Intertidal	MW817935	Yes	85%, *B. jacksonianum*	19-08-2009	Erin Grey
Bsp6	*Botrylloides* sp.	Bahamas	Exact location unknown	Unknown	NA			01-09-1993	Sarah Cohen
Bsp7	*Botrylloides* sp.	Barnes Key, FL, USA	24° 56′ 5″ N 80° 47′ 5″ W	Seagrass beds (*Thalassia testudinum*)	NA			10-1999	Tom Frankovich
Bsp8	*Botrylloides* sp.	Barnes Key, FL, USA	24° 56′ 5″ N 80° 47′ 5″ W	Seagrass beds (*Thalassia testudinum*)	MT232730	Yes	92%, *B. perspicuus*	10-1999	Tom Frankovich
Bsp9	*Botrylloides* sp.	Barnes Key, FL, USA	24° 56′ 5″ N 80° 47′ 5″ W	Seagrass beds (*Thalassia testudinum*)	MW817939	Yes	91%, *B. perspicuus*	10-1999	Tom Frankovich
Bsp10	*Botrylloides* sp.	Colon, Panama	9° 21′ 57.6″ N 79° 54′ 05.8″ W	Unknown	MT232729	No	89%, *B. simodensis*	22-03-2007	Greg Ruiz
Bsp11	*Botrylloides* sp.	Bocas del Toro, Panama	9° 20′ 07.1″ N 82° 14′ 46.1″ W	Docks	MH122634	No	89%, *B. simodensis*	01-07-2017	Marie Nydam
Bsp12	*Botrylloides* sp.	Bocas del Toro, Panama	9° 20′ 07.1″ N 82 °14′ 46.1″ W	Docks	MH122634	No	89%, *B. simodensis*	03-07-2017	Marie Nydam
Bsp13	*Botrylloides* sp.	Bocas del Toro, Panama	9° 20′ 07.1″ N 82° 14′ 46.1″ W	Docks	MH122634	No	89%, *B. simodensis*	03-07-2017	Marie Nydam
Bsp14	*Botrylloides* sp.	Burraneer Bay, Sydney, Australia	34° 03′ 37.7″ S 151° 08′ 07.1″ E	Unknown	MT232724	Yes	86%, *Botryllus* sp.	22-01-2008	Derrick Cruz
Bn1	*Botrylloides niger*	Honolulu, HI	21° 22′ 14.6″ N 157° 56′ 11.8″ W	Docks	MW817940	No	86%, *B. diegensis*	18-01-2012	Carrie Craig
Bn2	*Botrylloides niger*	Twin Cayes, Belize	16° 49′ 47.0″ N 88° 06′ 12.2″W	Dock, collected on PVC rack	KU711787.1	Yes	90%, *B. aster*	06-07-2007	Kristen Larson
Bn3	*Botrylloides niger*	Bocas del Toro, Panama	9° 20′ 14.8″ N 82° 10′ 38.2″ W	Mangrove roots	MT232728	No	90%, *B. arenaceus*	22-06-2017	Marie Nydam
Bn4	*Botrylloides niger*	Bocas del Toro, Panama	9° 20′ 14.8″ N 82° 10′ 38.2″ W	Mangrove roots	MT232723	No	90%, *B. arenaceus*	22-06-2017	Marie Nydam
Bd1	*Botrylloides diegensis*	Nomi-Wan, Kochi Prefecture, Japan	33° 21′ 28″ N, 133° 18′ 36″ E	Intertidal	NA			16-02-2005	Shinsuke Saito
Bd2	*Botrylloides diegensis*	Shizugawa, Japan	38° 38′ 41.2″ N 141° 28′ 34.6″ E	Docks	MT232722	No	86%, *Botryllus* sp.	30-06-2009	Erin Grey
Bd3	*Botrylloides diegensis*	Shimoda, Japan	34° 39′ 57.7″ N 138° 56′ 15.1″ E	Rocky Intertidal	MW817941	Yes	87%, *B. niger*	01-04-2007	Yas Saito
Bd4	*Botrylloides diegensis*	Sausalito, CA, USA	37° 51′ 45.4″ N 122° 29′ 18.7″ W	Docks	MN175981.1	No	86%, *B. niger*	28-09-2016	Marie Nydam
Bd5	*Botrylloides diegensis*	Long Beach, CA, USA	33° 43′ 05.1″ N 118° 16′ 44.1″ W	Docks	MN175984.1	No	86%, *B. niger*	20-02-2003	Sarah Cohen
Bd6	*Botrylloides diegensis*	Pelorus Sound, NZ	41° 08′ 50.3″ S 173° 51′ 50.7″ E	Mussel aquaculture	MW817942	Yes	87%, *B. niger*	01-04-2012	Gretchen Lambert
Bd7	*Botrylloides diegensis*	Port Nelson, New Zealand	41° 15′ 41.4″ S 173° 16′ 58.0″ E	Docks	MW817943	Yes	87%, *B. niger*	01-04-2012	Gretchen Lambert
Bd8	*Botrylloides diegensis*	Port Nelson, New Zealand	41° 15′ 41.4″ S 173° 16′ 58.0″ E	Docks	MN175987.1	No	86%, *B. niger*	01-04-2012	Gretchen Lambert
Bsp15	*Botrylloides* sp.	Medio Island, Philippines	13° 31′ 30.7″ N 120° 56′ 50.5″ E	Coral Reef	NA			16-04-2015	Sarah Cohen
Bsp16	*Botrylloides* sp.	Manila Channel, Puerto Galera, Philippines	13° 31′ 13.44″ N 120° 57′ 5.04″ E	Coral Reef	NA			19-04-2015	Sarah Cohen
Bsp17	*Botrylloides* sp.	Batangas Channel, Puerto Galera, Philippines	13° 31′ 16.788″ N 120° 57′ 34.74″ E	Coral Reef	NA			01-05-2015	Sarah Cohen
Bsp18	*Botrylloides* sp.	Batangas Channel, Puerto Galera, Philippines	13° 31′ 16.788″ N 120° 57′ 34.74″ E	Coral Reef	NA			29-04-2015	Sarah Cohen
Bsp19	*Botrylloides* sp.	Maricaban Island, Philippines	13° 45′ 34.272″ N 120° 55′ 34.104″ E	Coral Reef	NA			01-05-2014	Sarah Cohen
Bsp20	*Botrylloides* sp.	Maricaban Island, Philippines	13° 41′ 3.408″ N 120° 49′ 48.864″ E	Coral Reef	MW817938	Yes	84%, *B. schlosseri*	26-04-2014	Sarah Cohen
Bsp21	*Botrylloides* sp.	Maricaban Island, Philippines	13° 40′ 23.8″ N 120° 50′ 32.1″ E	Coral Reef	MW817937	Yes	84%, *B. schlosseri*	30-04-2014	Sarah Cohen
Bsp22	*Botrylloides* sp.	Maricaban Island, Philippines	13° 40′ 16.86″ N 120° 50′ 43.368″ E	Coral Reef	NA			01-05-2014	Sarah Cohen
Bsp23	*Botrylloides* sp.	Maricaban Island, Philippines	13° 41′ 15.144″ N 120° 50′ 28.068″ E	Coral Reef	NA			30-04-2014	Sarah Cohen

### Phylogenetic relationships among botryllid species

200 Anchored Hybrid Enrichment (AHE) loci were sequenced in 55 specimens. The mean length of the loci is 705 bp and the median length is 296 bp, indicating that the distribution of locus length is skewed to the right. The range of locus length is between 121 and 5172 bp. The total length of the alignment is 141,107 bp.

**Figure 1:**
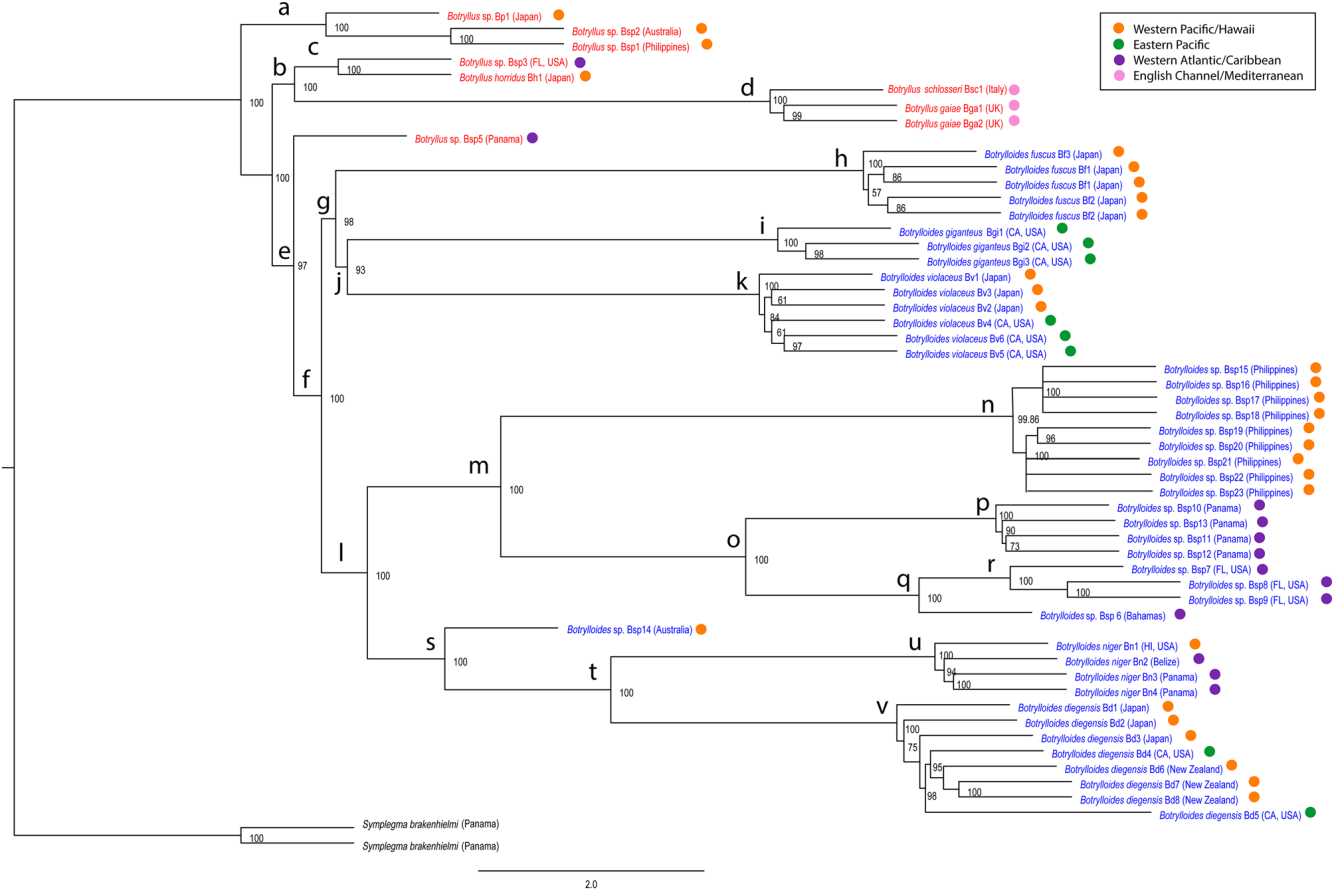
Maximum Quartet Support Species Tree of relationships among botryllid species, using the program Accurate Species TRee ALgorithm (ASTRAL). Nodes with less than 50% posterior probability support have been collapsed. The scale bar indicates two coalescent units. Red text = Genus *Botryllus,* Blue text = Genus *Botrylloides.* Colored circles refer to geographic regions (Orange = Western Pacific/Hawaii, Green = Eastern Pacific, Purple = Western Atlantic/Caribbean, Pink = English Channel/Mediterranean).

Figure 1 is the tree generated by ASTRAL, and Supplementary Fig. S3 is the tree generated by RAxML. The topologies of the two trees are identical. The *Botryllus* genus is paraphyletic with respect to the *Botrylloides* genus. Focusing on the *Botryllus* genus first, there are three *Botryllus-*only clades (a, c, and d in Fig. 1), Clade a comprising Botryllus sp. (Bp1), and two specimens from the Western Pacific (the Philippines and Australia: Bsp1 and Bsp2). Clade c includes a *Botryllus* specimen from Florida (Bsp3) and a specimen of *Botryllus horridus* from Japan (Bh1). Clade d is formed by *Botryllus gaiae* (Bga1, Bga2), and *Botryllus schlosseri* from Italy (Bsp4), and is a sister group to Clade c. A specimen we collected in Bocas del Drago (Bsp5), Panama (in the Bocas del Toro archipelago), is the only *Botryllus* in a clade (e) that includes all the *Botrylloides* species. We will refer to this specimen as Bocas del Drago.

Moving on to the *Botrylloides* portion of the phylogeny, Clade g contains *Botrylloides fuscus* (Bf1–Bf3 in Clade h) as a sister taxon to a *Botrylloides giganteus/Botrylloides violaceus* group (Clade j). Clade r contains three specimens (Bsp7–9) of a species we are calling Rabbit Key in this manuscript, based on its collection location offshore of Rabbit Key in the Florida Keys. This species has also been found offshore of Barnes Key in the Florida Keys (the location of the sample in the phylogenomic trees). Clade r is a sister clade to Clade q, containing a *Botrylloides* sp. from the Bahamas (Bsp6). Clade r + q is a sister group to Clade p, containing specimens (Bsp10–13) from a species found in Bocas del Toro, Panama, which we will refer to as Bocas del Toro. Clades p, q, and r form Clade o, which is a sister clade to Clade n, a species from the Verde Island Passage in the Philippines which is represented by Bsp16–Bsp23. Clade s includes a specimen from the Pacific (Sydney, Australia: Bsp14) as a sister group to a clade labeled *Botrylloides diegensis* (v) + a clade labeled *Botrylloides niger* (u). The clade labeled *Botrylloides diegensis* (v) contains specimens with three different names: *Botrylloides diegensis* from California (Bd4 and Bd5), *Botrylloides leachii* from New Zealand (Bd6–8), and *Botrylloides praelongus* from Japan (Bd1–3). For brevity’s sake, we labeled *Botrylloides diegensis/leachii/praelongus* samples as *Botrylloides diegensis* in Fig. 1, Supplementary Figs. S3 and S4.

### Morphological analyses in the botryllid group

**Figure 2:**
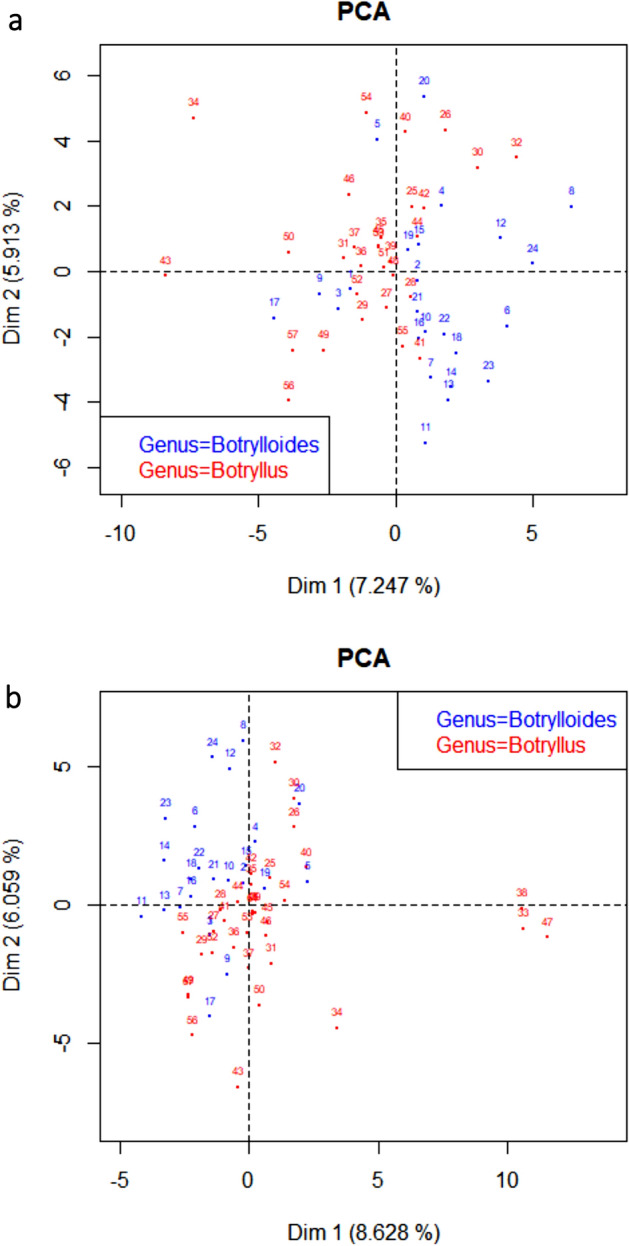
Graphical representation of Principal Components Analysis for morphological characters in botryllid species. The blue circles represent species in the genus *Botrylloides,* and the red circles species in the genus *Botryllus.* (**a**) Graph excluding three outlier species: *Botryllus flavus* (33), *Botryllus magnus* (38), and *Botryllus renierii* (47). (**b**) Graph including three outlier species: *Botryllus flavus* (33), *Botryllus magnus* (38), and *Botryllus renierii* (47). Species names corresponding to the numbers on the graphs in both (**a**) and (**b**) are as follows: (1) *Botrylloides anceps* (2) *Botrylloides aureus* (3) *Botrylloides chevalense* (4) *Botrylloides conchyliatus* (5) *Botrylloides crystallinus* (6) *Botrylloides diegensis* (7) *Botrylloides fuscus* (8) *Botrylloides giganteus* (9) *Botrylloides israeliense* (10) *Botrylloides leachii* (11) *Botrylloides lenis* (12) *Botrylloides lentus* (13) *Botrylloides magnicoecus* (14) *Botrylloides niger* (15) *Botrylloides perspicuus* (16) *Botrylloides praelongus* (17) *Botrylloides saccus* (18) *Botrylloides simodensis* (19) *Botrylloides superbus* (20) *Botrylloides tyreus* (21) *Botrylloides violaceus* (22) *Botrylloides* Bocas del Toro (23) *Botrylloides* Rabbit Key (24) *Botrylloides* Philippines (25) *Botryllus arenaceus* (26) *Botryllus aster* (27) *Botryllus closionis* (28) *Botryllus compositus* (29) *Botryllus delicatus* (30) *Botryllus eilatensis* (31) *Botryllus elegans* (32) *Botryllus firmus* (33)* Botryllus flavus* (34) *Botryllus gaiae* (35) *Botryllus gregalis* (36) *Botryllus horridus* (37) *Botryllus japonicus* (38) *Botryllus magnus* (39) *Botryllus meandricus* (40) *Botryllus mortenseni* (41) *Botryllus ovalis* (42) *Botryllus planus* (43) *Botryllus primigenus* (44) *Botryllus promiscuus* (45) *Botryllus pumilus* (46) *Botryllus puniceus* (47) *Botryllus renierii* (48) *Botryllus rosaceus* (49) *Botryllus scalaris* (50) *Botryllus schlosseri* (51) *Botryllus separatus* (52) *Botryllus sexiens* (53) *Botryllus stewartensis* (54) *Botryllus stuhlmanni* (55) *Botryllus tabori* (56) *Botryllus tuberatus* (57) *Botryllus* Bocas del Drago.

The results of the PCA analysis are presented in Fig. 2. All species have been given a numerical code (1–57), and the correspondence between the species name and the numerical code is presented in the legend. For easier viewing, Fig. 2a excludes three species that are widely divergent from the other botryllids: *Botryllus flavus, Botryllus magnus,* and *Botryllus renierii* (Species 33, 38, 47). These three outlier species, which are close together in the PCA, are included in Fig. 2b.

In general, *Botrylloides* species are morphologically similar to other *Botrylloides,* and the same for *Botryllus,* but there are several exceptions*.* There are 5 *Botrylloides* species that cluster with the *Botryllus* group: *Botrylloides anceps* (1),* Botrylloides chevalense* (3),* Botrylloides israeliense* (9), *Botrylloides saccus* (17), and *Botrylloides tyreus* (20). Four of these: *Botrylloides anceps* (1), *Botrylloides chevalense* (3), *Botrylloides israeliense* (9), and *Botrylloides tyreus* (20), were classified as *Botrylloides* based on a single character (the shape of the systems) when the majority of the characters are *Botryllus-*like^32,43–45^. The fifth species, *Botrylloides saccus* (17), was assigned to *Botrylloides* based on an assumption about brooding characters. However, the colony in question was not reproductively mature^46^. There are three *Botrylloides* that are positioned closely to the *Botryllus* cluster: *Botrylloides conchyliatus* (4), *Botrylloides perspicuus* (15), and *Botrylloides superbus* (19). There is one *Botryllus* that clusters with the *Botrylloides* group: *Botryllus ovalis* (41). The colonies have characteristics of both *Botrylloides* and *Botryllus*^47^. It should also be noted that the two clusters are close to each other in the PCA graph.

The majority of the nodes on the Bayesian phylogenetic tree were unresolved (i.e. polytomies), and the posterior probability support values for several resolved nodes were < 60 (Supplementary Fig. S5). The following clades were represented by nodes with clade credibility values > 60: *Botrylloides giganteus* (8) and *Botrylloides lentus* (12): clade credibility value = 86), *Botryllus magnus* (38) and *Botryllus renierii* (47): clade credibility value = 83), *Botrylloides magnicoecus* (13) and *Botryllus tabori* (55): clade credibility value = 79), and *Botryllus eilatensis* (30) and *Botryllus firmus* (32): clade credibility value = 82). These well-supported clades in the phylogenetic tree are consistent with the groupings in the PCA, although the clades do not include all species that are adjacent in the PCA. The undescribed species from the Philippines (24) is morphologically intermediate between *Botrylloides giganteus* (8) and *Botrylloides lentus* (12), but is not present in the phylogenetic clade comprising these two species. *Botrylloides fuscus* (7) and *Botryllus ovalis* (41) are intermediate to *Botrylloides magnicoecus* (13) and *Botryllus tabori* (55) in the PCA but are not in the *magnicoecus/tabori* clade, and *Botryllus flavus* (33) is intermediate to *Botryllus magnus* (38) and *Botryllus renierii* (47) but is not in the *magnus/renierii* clade. Only *Botryllus eilatensis* (30) and *Botryllus firmus* (32) are exclusive groups in both the PCA and the phylogenetic tree.

The distances between the species in Fig. 2 point to low morphological variation among many botryllid species. Despite this, there are clear morphological outliers. For example, *Botryllus primigenus* (43) and *Botryllus tuberatus* (56) both have small zooids (1.5 and 0.8 mm zooid length, respectively), where the average is 2.8 mm (Supplementary Table S1). *Botrylloides lenis* (11) has smaller zooids (1.3 mm, when the average is 2.2 mm: Supplementary Table S1) and a thinner tunic than other *Botrylloides* (1.7–2.0 mm, when the average is 3.25 mm: Supplementary Table S1), although external colony morphology such as tunic thickness may be environmentally influenced (Brunetti 2009, CS Cohen personal observation). *Botrylloides giganteus* (8) has the largest zooids in the botryllid group (18–20 stigmatal rows), where the average is 10–12. The tunic thickness in this species can reach 15 mm, when the average in the *Botrylloides* is 3.25 mm.

It should be noted that the first two principal components only represent 13% of the variation in the morphological characters when the outliers are excluded and 14% when they are included, so the attempt to reduce the dimensionality of the data set excluded a large proportion of the useful variation between the species. Therefore, the values in these characters cannot be easily correlated with one other.

## Discussion

The topology of the nuclear tree shown in Fig. 1 can be compared to the topologies of three previously published trees. The first two trees were constructed using 13 mitochondrial proteins^38,48^, and the third using 18 s rDNA^39^. The species represented in the mitochondrial trees are *Botryllus schlosseri*, *Botrylloides giganteus* (identified as *Botrylloides pizoni* in Ref.^48^), *Botrylloides leachii* (which may be *Botrylloides diegensis*^24^, *Botrylloides niger*, and *Botrylloides violaceus*. The more recent mitochondrial tree^38^ also includes *Botryllus gaiae*, which was formerly Clade E in the *Botryllus schlosseri* complex. In both of these mitochondrial trees, the *Botrylloides* clade is sister to the *Botryllus* clade. This is in contrast to Fig. 1, where *Botryllus* is paraphyletic with respect to *Botrylloides* although based on a single taxon (Bsp5). Within the *Botrylloides* clade, *Botrylloides leachii* and *Botrylloides niger* are sister species in both mitochondrial trees and in Fig. 1. *Botrylloides giganteus* and *Botrylloides violaceus* are sister species in Ref.^38^ and Fig. 1, whereas *Botrylloides violaceus* is sister to a *giganteus/leachii/niger* clade in Ref.^48^).

Comparing nodes in Fig. 1 to well-supported nodes in Ref.^39^’s 18 s rDNA Maximum Likelihood Tree (those nodes that have ≥ 80% bootstrap support), both trees show *Botryllus* species as the outgroups to the *Botrylloides* clade. The 18 s rDNA tree includes *Botrylloides fuscus*,* Botrylloides violaceus*,* Botrylloides simodensis*, and a sample from Venice Lagoon that was provided and named as *Botrylloides leachii* by A. Sabbadin. *Botrylloides fuscus* is an outgroup to a *violaceus*,* leachii*,* simodensis* clade, but the relationship is not well-supported. In the current phylogeny, *Botrylloides fuscus* is in a clade with *Botrylloides violaceus*, and this *Botrylloides fuscus/Botrylloides violaceus* clade is sister to a clade that includes *Botrylloides diegensis.*

Previous studies in the *Botryllus schlosseri* species complex have revealed that sister species can have very different dispersal patterns^42,49,50^. There are five clades in the species complex, with *Botryllus schlosseri* and *Botryllus gaiae* having widespread ranges, and Clades B, C, and D geographically restricted. Beyond the *Botryllus schlosseri* species complex, the four widespread species in the botryllid group are *Botrylloides diegensis*,* Botrylloides giganteus*,* Botrylloides niger*, and *Botrylloides violaceus. Botrylloides* species may have a higher likelihood of global spread than *Botryllus* species, despite *Botrylloides* being less diverse than *Botryllus* (21 vs. 32 described species). *Botrylloides* larvae are larger than *Botryllus* larvae and have longer developmental times^39^, which could impact dispersal or settlement abilities. In the current phylogeny, *Botrylloides giganteus* and *Botrylloides violaceus* are sister groups, as are *Botrylloides diegensis* and *Botrylloides niger* (Fig. 1 and Supplementary Fig. S3)*.* However, these sister groups may not remain as such when additional species are added, and testing whether range size has a phylogenetic signal across the entire group will require a more taxonomically comprehensive phylogeny.

The morphological distinctions between the genera *Botryllus* and *Botrylloides* have been much debated, and taxonomists have disagreed about whether there should be one genus or two genera (reviewed in Ref.^32^). Based on the morphological analyses in this study, species in each genus cluster together, although there is some overlap where the clusters meet. The five *Botrylloides* species that cluster with *Botryllus* in the PCA were named for a single *Botrylloides-*like character when the majority of the characters are *Botryllus-*like. This supports the argument that the two genera should be maintained^32^. But with a robust and broadly representative phylogeny, the generic assignations in this group can now be evaluated in a phylogenetic context.

According to phylogenetic systematics, all taxa should be monophyletic^51^. While some evolutionary systematists have argued for the maintenance of paraphyletic groups^52,53^, the precise location in the phylogeny where a nested group should be given a different name cannot easily be determined because evolution is most often a gradual process^54,55^. For this reason and others detailed in Ref.^55^, a broad agreement in the systematic community has formed in favor of monophyly^55–57^.

We classified the Bocas del Drago specimen (Bsp5) as *Botryllus* because many of its morphological characters were *Botryllus*-like. This species falls at the base of the *Botrylloides* clade. In a two genera classification scheme, the *Botryllus* clade would comprise two monophyletic groups plus one species in the *Botrylloides* clade. This paraphyly of *Botryllus* is also reflected in the 18S rDNA phylogeny^39^. Because the genus *Botryllus* is not a monophyletic group, we suggest that the generic distinctions within the botryllid group be re-evaluated. Based on the molecular phylogeny presented here, we agree with taxonomists who propose that the genus *Botrylloides* should be deleted, as it is a junior synonym of the genus *Botryllus*^37,47^. Their argument is based on morphology: no clear morphological distinction exists between the two genera and all morphological characters represent “various states of continuous evolution”^37,47^.

The botryllid ascidians are taxonomically understudied, despite their research significance as model systems, and the extensive ranges of *Botrylloides diegensis*, *Botrylloides giganteus*, *Botrylloides niger*, *Botryllus schlosseri*, and *Botrylloides violaceus*. Several widely distributed botryllid ascidians have been misidentified, and correct identification of these species is critical for understanding their biology and spread as well as detecting the spread of additional species. The identification of botryllid species within each genus can be accomplished through morphological examination for those species that are clear morphological outliers. But the majority of the botryllid species are morphologically very similar to several other species, so discrimination based on morphological characters alone is very difficult. Species delimitation analyses are therefore necessary to identify and describe species in this group. While the current phylogenomic tree does not have enough samples per taxon to conduct such analyses, we can obtain preliminary results from mtCOI barcode sequences. Table 1 includes a column describing whether each barcode is unique (i.e. does not have 100% identity to any other sequences on GenBank). Table 1 also lists the best BLAST result where the subject and query are labeled as different species, to illustrate the amount of divergence between species. Not all barcodes are unique, but when a barcode query exhibit 100% identity, the subject is always the same species. When subject and query are labeled as different species, the % identity between them is 92% or lower, suggesting a barcode gap between species.

The mtCOI barcodes provide preliminary evidence that the barcoded taxa in the tree are distinct species. Robust species delimitation analyses will be possible using the 200 AHE loci developed here. These loci could also be used to expand the current phylogenomic tree, in order to fully represent the evolutionary relationships within the botryllid group.

## Methods

### Sample collection

Samples were collected from 1995 to 2017 from both artificial and natural substrates (Table 1). The collection location, geographic coordinates, habitat, and GenBank accession number of mitochondrial cytochrome oxidase I (mtCOI) gene for each sample are presented in Table 1. Photographs of samples can be viewed in Supplementary Fig. S4. A small piece of tissue was removed from each colony in the field, cleaned to remove algae and other contaminants, and preserved in 95% ethanol, RNAlater (Thermo-Fisher), or a DMSO solution saturated with NaCl. For the species that are described in a morphological context in this study, colonies were relaxed using menthol crystals and subsequently preserved in 10% formalin in salt water buffered with sodium borate. Some samples collected in the Philippines were relaxed with tricaine methanesulfonate (MS222). However, we do not recommend this for ethanol samples that are intended for genetic analysis; these samples required multiple rounds of isopropanol precipitation at the end of the DNA extraction process in order to purify them, and even with purification did not always produce usable libraries.

### Sample identification: molecular techniques

We assigned samples to species by sequencing the mitochondrial cytochrome oxidase I (mtCOI) gene (Table 1). DNA was extracted using the Nucleospin Tissue Kit (Macherey Nagel). DNA was initially extracted from pieces of whole colony for each sample. In cases where extracted DNA failed to amplify, DNA was then extracted from zooids that were dissected from the colony. PCR amplification was performed using either OneTaq DNA Polymerase (New England Biolabs) or Phusion High-Fidelity DNA Polymerase (New England Biolabs). OneTaq reactions were as follows: 20 µl total reaction volume with 2 mM MgCl_2_, 0.2 mM dNTPs, 2 µl of 10× buffer, 0.2 mM of each primer, and 0.16 U of OneTaq. Phusion reactions were as follows: 20 µl total reaction volume with 4 µl HF buffer, 0.2 mM dNTPs, 0.6 µl of 100% DMSO, 0.2 U of Phusion. The amount of water and template DNA was individually determined for each PCR reaction, based on the concentration of template DNA in the sample: at least 30 ng of DNA was added to each PCR reaction.

Each DNA sample was amplified with one of two PCR primer pairs: Tun_forward/Tun_reverse2^58^, or LCO1490/HC02198^59^. Tun primers were only used with OneTaq polymerase, using this protocol: 94 °C for 1 min, 60× (94 °C for 10 s, 50 °C for 30 s, 72 °C for 50 s), 72 °C for 10 min. Folmer primers were only used with Phusion polymerase, using this protocol: 98 °C for 30 s, 35× (98 °C for 10 s, 48 °C for 30 s, 72 °C for 30 s), 72 °C for 5 min. PCR products were incubated with 1 µl each of Exonuclease I (New England Biolabs) and Antarctic Phosphatase (New England Biolabs) at 37 °C for 1 h, followed by 90 °C for 10 min. The PCR products were sequenced at the University of Kentucky's HealthCare Genomics Core Laboratory using an ABI-3730 automated sequencer (Applied Biosystems). Forward and reverse sequences were edited and combined into a consensus sequence using Codon Codes Aligner (Codon Code Corporation).

We compared each sequence to botryllid sequences available on GenBank. If the sequence we obtained had 98–100% identity to a sequence identified on GenBank using blastn, we considered our sample to be the same species as the sample on GenBank. Because GenBank sequences can be mis-assigned, we only used GenBank identifications in which the submitting author had independently verified the taxonomic assignment of the sample using morphological characters. In many cases, the mtCOI sequence had no close match on GenBank.

### Sample identification: morphological techniques

Taxa were also assigned to species by examination of morphological characters, when formalin preserved samples were available, using descriptions from the literature^11,30–36,38,40,43–48,60–65^. The 31 analyzed morphological characters are summarized as follows: arrangement of systems, position of ovaries and testes, testes morphology, number of stigmatal rows, completeness of the second stigmatal row, arrangement of stigmata, shape of intestine, location of anterior edge of intestinal loop, location of anus, number of stomach folds, appearance of the stomach folds, shape of the stomach, shape and size of the pyloric caecum, number and size orders of the oral tentacles, distribution of pigment cells in the zooid, zooid length, colony color when living and after fixation, and tunic thickness^30–32^. If the species could not be identified with complete certainty from the literature, we used a subset of the 31 characters: arrangement of systems, position of ovaries and testes (if ovaries and testes were present), appearance of the stomach folds, shape of the stomach, and size of the pyloric caecum. At least 30 zooids were examined from each colony.

When possible, we examined the morphology of the same colony from which the mtCOI sequencing was obtained. If a formalin preserved sample of the original colony was not available, we were often able to examine the morphology of another colony with an identical mtCOI sequence. We did not assign a specimen to a specific morphologically-described species if we could not obtain a formalin sample of the original colony or of a colony with an identical mtCOI sequence.

In the course of our species assignment using morphology, several specimens did not match the descriptions of any species previously found in the geographic region in which the specimen was collected. Either these samples represent new species, or they are known species that have only recently been identified from the location where they were collected. To determine where these species fit in relation to the morphologies of the other *Botryllus/Botrylloides* species, we searched the literature for morphological information on the 53 described botryllid species. We compiled data on the 31 morphological characters described above. The genus names (*Botrylloides* or *Botryllus*) and the morphological data come from the type description. In a few cases, the type description is lacking data on the majority of these characters. If a re-description was available, it was used to supplement the type description. Because our examinations were more thorough than type descriptions (often type descriptions do not provide data on individual zooids or individual colonies), we averaged our quantitative data across the 30 + zooids we examined in each colony to obtain a single value for each colony. We then averaged quantitative data across multiple colonies to obtain a single value for each character for each species, to match the data available in the literature. A brief description of each considered morphological character is available in Supplementary Fig. S2, and the entire data matrix is available in Supplementary Table S1. Using the morphological data from the 53 described species and the 4 undescribed species, we then conducted a Principal Components Analysis using PCAmixdata^66^ as implemented in R version 3.6.1.

To accompany the PCA, we constructed a phylogenetic tree of the 57 species using the 31 morphological characters using MrBayes 3.2.2^67^ on the CIPRES (Cyberinfrastructure for Phylogenic Research) Science Gateway^68^. The GTR + G model of nucleotide substitution was applied to all data sets (Nset = 6). Each analysis was run for 10 million generations, with sampling every 1000 generations. The first 2000 trees were eliminated as burn-in.

### Anchored hybrid enrichment (AHE) locus identification and probe design

Our aim was to develop a resource for collecting hundreds of orthologous loci across the botryllid ascidians using Anchored Hybrid Enrichment (AHE)^69^. The pre-existing genomic resources included an assembled genome of *Botryllus schlosseri*^70^, and two assembled transcriptomes: *Botryllus schlosseri*^71^, and *Botrylloides leachii*^72^, recently re-assigned to *Botrylloides diegensis* in Ref.^24^. In order to better represent the high diversity of the botryllid group, we collected low-coverage, whole genome data assemblies for seven additional species (details are given in Supplementary Table S2). DNA extracts for these seven species were sent to the Center for Anchored Phylogenomics (http://www.anchoredphylogeny.com) for processing. In brief, after the quality/quantity of DNA was assessed using Qubit, Illumina libraries with single 8 bp indexes were prepared following^73^, with modifications described in Ref.^74^. Libraries were pooled and sequenced on two Illumina HiSeq2500 lanes with a paired-end 150 bp protocol. A total of 125 Gb of data was collected yielding 25–65 × coverage per species. Reads were filtered for quality using the Cassava high chastity filter, demultiplexed with no mismatches tolerated, and merged to remove sequence adapters^75^ prior to downstream processing.

In order to identify suitable conserved targets for AHE, we performed reciprocal blast on local machines at the Center for Anchored Phylogenomics using the two assembled transcriptomes (blastn). Using the results from the blast searches, we identified 482 preliminary targets with matching transcripts, which we aligned using MAFFT v7.023b^76^. Alignments were manually inspected in Geneious (vR9, Biomatters Ltd., Kearse et al. 2012), then trimmed to regions that were well-aligned. For the remainder of the locus development/identification, we followed the protocol outlined in Ref.^77^. More specifically, we isolated the *Botryllus schlosseri* (transcriptome) sequences from the aforementioned alignments, and using those as a reference scanned the *Botryllus schlosseri* genome for the AHE regions. Regions of 10,000 bp containing a 17 of 20 initial spaced k-mer match, followed by a 55 of 100 confirmation match to one of the references were kept. K-mers are all of a sequence’s subsequences of length = k. For example, the sequence GCTA would have the following k-mers: G, C, T, A, GC, CT, TA, GCT, CTA, and GCTA. K-mers from the *Botryllus schlosseri* transcriptome were used to search the *Botryllus schlosseri* genome for AHE regions, and matches were based on spaced seeds as described in Ref.^78^. We then aligned (using MAFFT), the best matching genome sequence for each locus to the two transcriptome-derived sequences for that locus. Using Geneious (vR9, Biomatters Ltd.), we identified well-aligned regions of each three-sequence alignment and trimmed the alignments accordingly. The three-sequence alignment contained only two species: *Botryllus schlosseri* and *Botrylloides leachii* (recently re-assigned as *Botrylloides diegensis*)^24^.

In order to incorporate whole genome sequencing (WGS) data from the seven additional species, we utilized sequences from *Botrylloides leachii* and *Botryllus schlosseri* in the alignments as references. Each WGS read was checked against the reference database and reads with a preliminary 17 of 20 initial spaced k-mer match, followed by a final 55 of 100 bp consecutive match were retained, then aligned by locus to form seeds for an extension assembly that allowed flanking regions to be recovered (see Ref.^77^ for details and scripts). In order to construct the final alignments, the (up to) 10 sequences for each locus were aligned in MAFFT, then trimmed to well-aligned regions after inspection in Geneious (vR9, Biomatters Ltd.). In order to avoid problems associated with missing data in downstream projects^79^, loci represented by less than 50% of the sequences in the alignment were removed from downstream analysis. When alignments from two loci were found to be overlapping (i.e. containing some of the same 20-mers), one locus was removed to ensure that each locus was a unique target. Lastly, we checked for repetitive elements by profiling the k-mers found in the alignments with respect to their occurrence in the WGS reads. Regions with a substantially elevated k-mer coverage were masked. A total of 200 AHE targets resulted from the process. Supplementary Table S3 contains the size of each locus, and genomic position of each locus in the *Botryllus schlosseri* genome, as determined from the best blastn match to the *Botryllus schlosseri* genome assembly using the locus sequence as a query. Finally, in-silico probes were tiled uniformly across the 10 sequences for each locus at 3.5 × coverage depth. A total of 54,350 probes covered the 200 AHE targets (total target size ~ 139 kb) that resulted from the process. These loci were successfully amplified in *Symplegma brakenhielmi*, to provide an outgroup for the phylogenomic tree. These loci will therefore be useful for *Symplegma*, which is the sister group to the *Botrylloides/Botryllus clade*^80^. The utility of these loci beyond the genera *Botrylloides*, *Botryllus* and *Symplegma* has not been investigated.

### DNA extraction and library preparation methods

DNA for all samples in Table 1 was extracted using the E.Z.N.A DNA isolation kit (Omega BioTek), and an additional isopropanol precipitation was performed to further purify the DNA. The quantity and quality of the DNA extractions were determined using Qubit and 2% TAE agarose gels. The extracted DNA was fragmented into 300–500 bp pieces using a Covaris E220 focused-ultrasonicator with microTUBES (Covaris). Then, library preparation and indexing were performed on a Beckman-Coulter Biomek FXp liquid-handling robot, using a protocol based on Ref.^73^. Anchored hybrid enrichment was performed using a custom SureSelect kit (Agilent Technologies) targeting loci designed from the whole genome alignment. Sequencing data were generated on an Illumina HiSeq2500 platform at the Center for Anchored Phylogenomics at Florida State University (www.anchoredphylogeny.com), as in Ref.^79^. Sequencing was performed in the Translational Science Laboratory in the College of Medicine at Florida State University.

### Raw read alignment

Sequence reads were demultiplexed with no mismatches tolerated and filtered for quality using the Illumina CASAVA pipeline with a high chastity setting. Overlapping reads were identified and merged using the approach described by Ref.^75^. This process removes sequence adapters and corrects sequencing errors in overlapping regions. Reads were then assembled using the quasi-de novo approach described by Ref.^77^. This assembly approach uses divergent references to identify sequences coming from conserved regions to which reads can be mapped. The mapped reads are in turn used as references when the assembly is extended into less conserved regions (see Ref.^77^ for details). Probe region sequences from eight of the nine species used in the probe design were used as references for the initial mapping, while sequences from the *Botryllus schlosseri* genome (the 9th species) served as the primary reference. Consensus sequences were constructed from assembly clusters containing greater than an average of 250 reads. Ambiguity codes were employed for sites in which base frequencies could not be explained by a 1% sequencing error.

### Phylogenomic tree building

Orthologous groups of consensus sequences were identified using a clustering approach that relied on an alignment-free distance matrix constructed by measuring the degree of 20-mer distribution overlap among taxa (see Ref.^77^ for details). Sequences from orthologous sets of loci were then aligned using MAFFT (v7.023b^76^). Alignments were trimmed and masked (i.e. excluded) using the automated procedure described by Ref.^77^, with 50% threshold required for identifying reliable sites, a 14-base threshold for masking misaligned regions, and 25 sequences required to be present at a site to prevent removal of the site. Alignments were inspected visually in Geneious (vR9, Biomatters Ltd.) to ensure that the settings used in the automated procedure were appropriate and also to identify any undetected misaligned regions.

Phylogenomic trees were built using two methods: a concatenated species tree method using RAxML v8.2.8^81^, and a coalescent species tree method using ASTRAL-II v4.10.12^82^. Maximum likelihood gene trees were first created for each locus separately. Then, maximum likelihood trees were created using a concatenated alignment partitioned by locus. A GTR + G model of nucleotide substitution and 1000 bootstrap replicates were employed for both gene trees and the species tree. The gene trees produced by RAxML were then used as inputs for ASTRAL-II. ASTRAL-II obtains quartet trees from the gene tree inputs, and creates a species tree that contains the maximum number of quartet trees present in all gene trees^82^.

A maximum likelihood tree-building framework using concatenated multiple gene alignments to obtain a species tree is a common approach^83,84^. We also employed a second, coalescent-based method (ASTRAL-II). Coalescent-based methods are often used because concatenation can lead to inaccurate species trees with high levels of bootstrap support^84^. ASTRAL-II is a summary method^83^, and is preferable to Bayesian co-estimation coalescent methods due to computational difficulties with datasets containing > 100 loci or > 30 samples^85–87^.

## Supplementary Information

Supplementary Figures.

Supplementary Table S1.

Supplementary Table S2.

Supplementary Table S3.

## Data Availability

All mtCOI barcode sequences associated with this study have been uploaded to GenBank: Accession numbers are available in Table 1. Genome raw reads, genome assemblies, and alignments for probes are available on Dryad (https://doi.org/10.5061/dryad.3r2280gf7). All other data generated or analyzed during this study are included in this published article (and its Supplementary Information files).
