# Supplementary material for: Phylogenomic and morphological relationships among the botryllid ascidians (Subphylum Tunicata, Class Ascidiacea, Family Styelidae)
Source: Sci Rep. 2021 Apr 16;11:8351. doi: 10.1038/s41598-021-87255-2 (PMC8052435; doi:10.1038/s41598-021-87255-2)
Supplement: Supplementary file 1 — Supplementary Figures. [file 41598_2021_87255_MOESM1_ESM.pdf]

Phylogenomic and morphological relationships among the botryllid ascidians (Subphylum Tunicata, Class Ascidiacea, Family Styelidae)

Marie L. Nydam[1,*], Alan R. Lemmon[2], Jesse R. Cherry[3], Michelle L. Kortyna[3], Darragh L. Clancy[4], Cecilia Hernandez[4], C. Sarah Cohen[4]

* Corresponding Author: mnydam@soka.edu

[1] Math and Science Program, Soka University of America, 1 University Drive, Aliso Viejo, CA 92656, USA

[2] Department of Scientific Computing, Florida State University, 400 Dirac Science Library, Tallahassee, FL 32306, USA

[3] Department of Biological Science, Florida State University, 319 Stadium Drive, Tallahassee, FL 32306, USA

[4] Biology Department and Estuarine and Ocean Science Center, San Francisco State University, 3150 Paradise Drive, Tiburon CA, 94920, USA

Supplementary Figure S1: Detailed morphological descriptions of each species, including zooid and colony pigmentation after formalin preservation, appearance and location of the ovaries and testes, stigmatal characters, intestine and stomach characters, and number and size of oral tentacles.

Morphological description: Bocas del Drago species

Photographs of this species can be found in Supplementary Fig. S4, and a complete morphological description can be found in Supplementary Fig. S2. This species has a single specimen in the phylogenomic tree, (Bsp5), and the species is labeled 57 in Fig. 2. There are only three botryllid species that have been described from the Caribbean: *Botrylloides magnicoecus, Botrylloides niger,* and *Botryllus planus.* If this species has been described in the literature, it is either *Botryllus planus* or *Botrylloides magnicoecus*. The descriptions of *Botryllus planus* vary considerably by geographic location: Brazil [48], Guadeloupe and Bermuda [49-50], and Bermuda, Curacao, Florida [11,51-52]. For example, Van Name draws the 2nd stigmatal row complete whereas Monniot draws it incomplete, Van Name describes 11-13 stigmatal rows, whereas Monniot describes 8, Van Name describes 9-10 stomach folds, Monniot describes 7-9, Van Name describes colonies as darkly colored, Monniot describes colonies as dark beige. Because the variation in these characters is broader than intraspecific variation in other botryllid species, these authors are very likely identifying more than one species as *Botryllus planus.* Of all the *Botryllus planus* descriptions, the colony we collected from Bocas del Drago matches most closely with Monniot's specimens from Guadeloupe and Bermuda [49-50].

According to Monniot [50], *Botrylloides magnicoecus* is a species or species complex with a very broad global distribution. Again, as characters in this species vary geographically, we will use the description of *Botrylloides magnicoecus* from the Caribbean [49-50]. Monniot says the distinguishing characteristics of the species are: 1) the barrel stomach with folds of the same width from the cardia to the pylorus 2) a very large caecum, and 3) an ovary dorsal to the testis. The stomach shape in the present specimen is similar to that of both *Botryllus planus* and *Botrylloides magnicoecus.* The caecum is very large in all three species, although the Bocas del Drago caecum is shaped more like the *Botryllus planus* than the *Botrylloides magnicoecus* caecum. This Bocas del Drago species has the ovary located anterior to testis, as does *Botryllus planus* but not *Botrylloides. magnicoecus*. In Fig. 2, the Bocas del Drago specimen is very close to *Botryllus planus* in morphological space, but far from *Botrylloides magnicoecus.* Given these characteristics, the Bocas del Drago specimen is most likely to be *Botryllus planus,* if it is a described species. However, identification of this species is dependent on taxonomic revisions of *Botrylloides magnicoecus* and *Botryllus planus.*

Photographs of this species can be found in Supplementary Fig. S4, Plate 7. A single colony was found in Bocas del Drago, Panama, in coral rubble. The colony is thin, not more than 1 mm thick. The zooids are small, on average 0.88 mm. The zooids are arranged in a "leachii-type" configuration (as described in Brunetti 2009): not all dorsal lips are involved in forming the cloacal opening (Plate 7a and 7b). In life the colony appears yellow, owing to yellow pigmentation in a partial ring around the dorsal side of the oral siphon (Plate 7a and 7b). Two black pigment clusters are present on either side of the pigmentation ring, just posterior to the oral siphon (Plate 7b). Zooids are covered densely in white pigment cells.

After formalin preservation, the zooids are peach-colored and opaque, such that internal organs are not visible from the outside of the zooid. The tunic is covered in purple pigment cells with moderate density, sometimes concentrated on the dorsal lip. Clusters of orange pigment cells are present on either side of the endostyle. In other species, each cluster is confined to a stigmatal row, but in these zooids the clusters are so large that they form a continuous line of pigment of either side of the endostyle. Purple pigment cells can be found in the transverse vessels as well (Plate 7d).

The ovary is anterior to the testis. The left side testis is just anterior to the anterior-most intestinal loop, and the posterior portion of the right testis is overlapping with the cardiac ends of the anterior-most stomach folds (Plate 7c). Therefore, the left side testis is anterior to the right side testis. The testis has two to six lobes (most commonly four), often in a rosette orientation (Plate 7c). Each zooid has eight rows of stigmata, with the second stigmatal row dorsally incomplete. There are 13 stigmata per half-row, three between the dorsal lamina and the first longitudinal vessel, three between the first and second longitudinal vessels, three between the second and third longitudinal vessels, and four between the third longitudinal vessel and the endostyle (DL 3 3 3 4 E).

The intestine is angular (base of the rectum and the intestine make an acute angle) (Plate 7e). The anterior edge of the intestinal loop extends to the 7th stigmatal row. The anus opens between the 4th transverse vessel and the 7th stigmatal row, most commonly at the 6th transverse vessel or the 7th row. The anus opens mostly commonly 1.5 stigmatal rows anterior to the anterior edge of the intestinal loop. The anus is bilobed. There are approximately eight stomach folds, without longitudinal grooves. The stomach is cylindrical, with no swellings on the cardiac ends of the stomach folds. The tip of the pyloric caecum bends at a right angle with respect to the base, and swells at the tip (Plate 7e). The pyloric caecum is large, would be nearly the length of the stomach if the tip were not bent (Plate 7e). The oral tentacles are of two size orders, with one of the large tentacles longer than the other three (denoted with an asterisk): L* S L S L S L S. The oral tentacles can be concave at the tips (so that the tentacles are shaped like spoons), and the large oral tentacles are often curved over at the tips.

<u>Morphological description: Bocas del Toro species</u>

Photographs of this species can be found in Supplementary Fig. S4, and a complete morphological description can be found in Supplementary Fig. S2. This species has four specimens in the phylogenomic tree, (Bsp10-13), and the species is labeled 22 in Fig. 2. This species is not likely to be native to the Bocas del Toro region, or to the Caribbean, given its prominence in marinas and mangroves adjacent to marinas. The physical appearance of the zooids in this species is similar to *Botrylloides niger* zooids*,* with a slight difference in the shape of the stomach: the pyloric end of the stomach is wider and the stomach folds are shorter in the Bocas del Toro species than in *Botrylloides niger.* However, the barcode sequences between the Bocas del Toro species and *Botrylloides niger* have 84% identity and the two species occupy distinct positions in the phylogeny (Fig. 1, Supplementary Fig. S3). In the PCA, this Bocas del Toro species clusters with six species that are morphologically very similar to each other: *Botrylloides diegensis, Botrylloides leachii, Botryllus ovalis, Botrylloides praelongus, Botrylloides simodensis,* and *Botrylloides violaceus* (Numbers 6,10,16,18,21,41 in Fig. 2).

Botrylloides diegensis and *Botrylloides leachii* have been confused in the literature and were only recently untangled using barcoding [23]. Five of these six species have barcodes, and the barcode of the Bocas del Toro species is distinct from all five (84-90% sequence identity between the barcode of the Bocas del Toro species and the other five species). The species without a barcode is *Botryllus ovalis.* This species was collected in New Caledonia, and is described by Monniot as very close to *Botrylloides niger* [47]. The only difference between the two species is in the form of the stomach, which is oval shaped in *Botryllus ovalis,* but trumpet-shaped in *Botrylloides niger* [47]. The shape of the *Botryllus ovalis* stomach is similar to the shape of the Bocas del Toro species. Identification of this Bocas del Toro species will depend on examination of the *Botryllus ovalis* type specimen.

Photographs of this species can be found in Supplementary Fig. S4, Plate 12. This species was common in Bocas del Toro, Panama. It was found on mangrove roots immediately adjacent to the boat dock at the Smithsonian Tropical Research Institute, on pilings in the Bocas del Toro Town and Red Frog marinas, and on mangrove roots immediately adjacent to these marinas. This species was not seen on the coral reefs. The colony is ~2 mm thick. The zooids are on average 1.82 mm long. The zooids are arranged in a "leachii-type" configuration (as described in Brunetti 2009): not all dorsal lips are involved in forming the cloacal opening (Plate 12g-i). We found three color morphs of this species: black and cream (Plate 12b-f), bright orange (Plate 12g-j), and dark orange (rust-colored) (Plate 12a,k). The bright orange morph (Plate 12g-j) can easily be mistaken for *Botrylloides niger,* which has a morph of the same color (Plate 13b-c). In the black and cream colonies, the majority of the zooid is black, with cream or yellow pigmentation forming a ring around the oral siphon, or covering the entire anterior portion of the zooid (Plate 12c-d). Because the zooids are arranged in rows, with the oral siphons of one row touching the oral siphons of the other, the colonies have meandering lines of cream coloration, and alternating lines of black coloration (Plate 12e-f). The ampullae on the edges of the colonies are generally cream. The bright orange zooids have higher concentrations of pigment cells in the anterior part of the zooid than in the posterior part of the zooid. The posterior part of the zooid has intersecting lines where the pigment cells are less dense; these lines have a grey color due to lack of pigment cells. The zooids of the dark orange morph have variable densities of pigment cells, but the higher density areas are not confined to the anterior portion of the zooid as in the bright orange zooids.

After formalin preservation, almost all of the zooids have dark purple pigment cells concentrated on the dorsal lip, in transverse vessels, and spread densely throughout the tunic. At least half of the zooids have concentrated dark purple pigment cells surrounding the dorsal rim of the oral siphon and large orange pigment clusters on either side of the endostyle, corresponding to stigmatal rows 2-7.

None of the zooids had ovaries, so we could not determine the position of the ovaries with respect to the testes. The left side testis is just anterior to the anterior-most intestinal loop, and the posterior portion of the right testis is overlapping with the cardiac ends of the anterior-most stomach folds. Therefore, the left side testis is anterior to the right side testis. Testis comprise 2-12 lobes, most commonly 6-7 (Plate 12n). When numerous, these lobes are not in a perfect rosette shape (e.g. lobes radiating out from a clearly defined center), but are clustered together in a sphere (Plate 12n). Each zooid has 10-12 rows of stigmata on each side, most commonly 11 (Plate 12l). The last row of stigmata can be irregular. The second

stigmatal row is dorsally incomplete, on both the left and right side of the zooid (Plate 12l). There are an average of 14.55 stigmata per half-row on the left side of the body, an average of 5.47 between the dorsal lamina and the first longitudinal vessel, 2.84 between the first and second longitudinal vessels, 2.52 between the second and third longitudinal vessels, and 3.72 between the third longitudinal vessel and the endostyle (DL 5.47 2.84 2.52 3.72 E). There are an average of 14.27 stigmata per half-row on the right side of the body, an average of 4.34 between the dorsal lamina and the first longitudinal vessel, 3.01 between the first and second longitudinal vessels, 2.89 between the second and third longitudinal vessels, and 4.03 between the third longitudinal vessel and the endostyle (DL 4.34 3.01 2.89 4.0 E)

The intestine is angular (base of the rectum and the intestine make an acute angle). The anterior edge of the intestinal loop extends to between the 7th and 11th stigmatal rows, with the 9th and 10th stigmatal rows being the most common. The anus opens between the 7th and 9th stigmatal rows, with the 8th row being the most common. The anus opens most commonly 1-1.5 stigmatal rows anterior to the anterior edge of the intestinal loop. The anus appears to have the same diameter all the way around (unilobed).

There are eight-nine stomach folds, without longitudinal grooves (Plate 12p). The stomach is cylindrical because the pyloric end isn't narrower than the cardiac end. However, the cardiac ends of the stomach folds have ovoid swellings (Plate 12o). The tip of the pyloric caecum does not bend at a right angle with respect to the base (Plate 12o). The tip of the pyloric caecum is moderately swollen (Plate 12o). The pyloric caecum is small (40% the length of the stomach) (Plate 12o). There are two or three size orders of eight oral tentacles, with the two sizes alternating as such: S L S L S L S L, and the three sizes alternating as such: L S M S L S M S.

<u>Morphological description: Rabbit Key species</u>

Photographs of this species can be found in Supplementary Fig. S4, and a complete morphological description can be found in Supplementary Fig. S2. This species has three specimens in the phylogenomic tree, (Bsp7-9), and the species is labeled 23 in Fig. 2. This species has been called *Botryllus planus* in a field guide [53]. However, the phylogenetic position of this species is deep within the *Botrylloides* clade. Morphologically, this species has characteristics associated with *Botrylloides,* including the arrangement of the systems, conical stomach, and small pyloric caecum. For these reasons, this species cannot be *Botryllus planus.* In the PCA, this species falls on the outer edge of the *Botrylloides* cluster, distinguished from the *Botryllus* cluster in both PCA Dimension 1 and Dimension 2 (Fig. 2). Its characteristics are similar to *Botrylloides fuscus, Botrylloides lenis, Botrylloides magnicoecus, Botrylloides niger,* and *Botrylloides simodensis* (Numbers 7,11,13,14,18 in Fig. 2). This species cannot be *Botrylloides magnicoecus* because the pyloric caeca are different sizes, and the barcode sequences and/or phylogenetic positions rule out the other four species. Despite the presence of this species in Florida for at least 30 years, this is likely to be an undescribed species that has been incorrectly identified as *Botryllus planus*.

Photographs of this species can be found in Supplementary Fig. S4, Plate 11. The single colony described here was found in eelgrass beds by Tom Frankovich offshore of Rabbit Key in

the Florida Keys. The colonies in the phylogeny are from nearby Barnes Key. This species has also been found in the Bahamas, based on identical barcodes (Xavier Turon, personal communication). The colony is 1-2 mm thick. The zooids are on average 1.5 mm long. The zooids are arranged in a "leachii-type" configuration: not all dorsal lips are involved in forming the cloacal opening. The color in life is grey, with yellow/gold pigmentation concentrated in between systems (Plate 11a). The ampullae are also yellow/gold.

When preserved, the colony takes on a deep purple color, although the previously yellow/gold pigmentation between the systems and in the ampullae is retained as pale yellow. Zooids in tunic appear brown. There is a darker brown ring around the oral siphon, and inside the darker brown ring (immediately surrounding the oral siphon) is a yellow ring. When zooids are removed from the tunic, dark purple pigment cells in transverse vessels can easily be seen.

None of the zooids had ovaries, so we could not determine the position of the ovaries with respect to the testes. The left side testis is just anterior to the anterior-most intestinal loop, and the posterior portion of the right testis is overlapping with the cardiac ends of the anterior-most stomach folds. Therefore, the left side testis is anterior to the right side testis. Testis comprise 4-11 lobes, most commonly 9-11. When numerous, these lobes are not in a perfect rosette shape (e.g. lobes radiating out from a clearly defined center), but are clustered together in a sphere. Zooids have 14 rows of stigmata on each side. The second stigmatal row is dorsally incomplete, on both the left and right side of the zooid. There are an average of 13.23 stigmata per half-row on the left side of the body, an average of 4.6 between the dorsal lamina and the first longitudinal vessel, 2.59 between the first and second longitudinal vessels, 2.05 between the second and third longitudinal vessels, and 4 between the third longitudinal vessel and the endostyle (DL 4.6 2.59 2.05 4 E). There are an average of 13.06 stigmata per half-row on the right side of the body, an average of 4.38 between the dorsal lamina and the first longitudinal vessel, 2.5 between the first and second longitudinal vessels, 2.33 between the second and third longitudinal vessels, and 3.86 between the third longitudinal vessel and the endostyle (DL 4.38 2.5 2.33 3.86 E).

The intestine is angular (base of the rectum and the intestine make an acute angle). The anterior edge of the intestinal loop extends to between the 9th stigmatal row and the 10th transverse vessel. The anus opens between the 7th and 8th transverse vessel. The anus opens most commonly 1-1.5 stigmatal rows anterior to the anterior edge of the intestinal loop.

There are eight stomach folds, without longitudinal grooves. The stomach is conical, meaning that the pyloric end is narrower than the cardiac end. The cardiac ends of the stomach folds have ovoid swellings. The tip of the pyloric caecum bends slightly with respect to the base. The tip of the pyloric caecum is slightly swollen. The pyloric caecum is small (40% the length of the stomach). The oral tentacles could not be counted in this colony.

Morphological description: Philippines species

Photographs of this species can be found in Supplementary Fig. S4, and a complete morphological description can be found in Supplementary Fig. S2. This species has nine specimens in the phylogenomic tree (Bsp15-23), and the species is labeled 24 in Fig. 2. This

species is found in the Indo-Pacific, which contains the highest number of ascidian species globally [54]. However, only ten *Botrylloides* species have been described from this area. It is likely that this species, and many others, have not been described yet. In the PCA, this species occupies a morphological space close to *Botrylloides lentus* and *Botrylloides giganteus.* These species have larger zooids than the species from the Philippines, and *Botrylloides giganteus* occupies an entirely different place within the botryllid phylogenetic tree than the Philippines species.

Photographs of this species can be found in Supplementary Fig. S4, Plates 15-21. We examined six colonies (Bsp15, Bsp16, Bsp17, Bsp18, Bsp20, Bsp21) collected from two different locations: Medio Island/Puerto Galera (Bsp15-18) and Maricaban Island (Bsp19-23), Mindanao, Philippines. These colonies were collected in coral reef habitats. Two of the colonies are 2-2.5 mm thick (Bsp17 and 21), while the others are 5-6 mm thick. Zooid length ranges from 1.49 mm to 2.57 mm, with the average length being 2.06 mm. The zooids are arranged in a "leachii-type" configuration: not all dorsal lips are involved in forming the cloacal opening. Color patterns in this species vary, when the colonies are photographed alive. Photographs of Bsp18 and Bsp20 are not available. In Bsp15, zooids in each system are spaced further apart from each other than is usual in botryllids (Plate 15b). The tunic in the spaces between zooids is brown, and the zooids themselves are beige or cream-colored (Plate 15b). Where zooids are absent, the tunic is filled with yellow/gold pigmentation clusters (Plate 15b). In Bsp16, the zooids are white on the dorsal surface, and black on the ventral surface (with the color transition occurring at the location of the oral siphon) (Plate 16b). White pigment cells fill the tunic where the zooids are absent (Plate 16b). The tunic outside of the systems, including the cloacal structures, is an orange color (Plate 16b). The zooids in the tunic of Bsp17 appear grey, with white pigment clusters covering each zooid (Plate 17). There are yellow/gold pigmentation clusters in portions of the colony where zooids are not present (as in Bsp15), and the ampullae are yellow/gold (Plate 17). In Bsp21, only 2 systems are visible in the photographs. The tunic is transparent, colored light green around the cloaca at the center of each system (Plate 19). Both white and pink pigment cell clusters cover the zooids in the tunic (Plate 19).

After formalin-preservation, the color patterns are more similar in the six colonies. In Bsp15 and Bsp16, tunic is not completely transparent, has a whitish cloudy appearance that gives a slight opacity (even where pigment clusters are absent). Zooids inside the tunic are dark magenta to magenta in color. Ampullae are peach or beige. In Bsp18, tunic is transparent or nearly so, and zooids are dark purple inside the tunic. The large, sausage-shaped ampullae surrounding the systems are dark purple or peach-colored, depending on their location in the colony. In Bsp17, the zooids inside the tunic and ampullae are brown. In Bsp20 and Bsp21, colony tunic is pinkish and opaque. Ampullae are brown or reddish-brown, depending on their location in the colony.

When zooids are removed from the tunic, purple or magenta pigment cells are spread across the surface of the zooids at variable densities in all colonies. Purple or magenta pigment cells are also frequently found inside the transverse vessels (e.g. Plate 16b). In Bsp20, purple pigment cells cluster at the anterior end of the endostyle, and purple pigment cells are found in a large cluster in the center of the dorsal lip. In Bsp15, magenta pigment cells are clustered along posterior of dorsal lamina, anus, posterior edge of atrial opening, dorsal lip and oral

siphon. Cells are only dense on the dorsal lip and around the oral siphon. There are two ventral-to-dorsal lines of magenta pigment cells running from the oral siphon to the edges of the dorsal lip. In Bsp16, magenta pigment cells are clustered along the posterior edge of atrial opening, in an anterior/posterior line on the left and right edges of the tunic, closest to where the tunic meets the atrial opening, around the dorsal edge of the dorsal lip and around the oral siphon (Plate 16b). Orange pigment clusters are present on either side of endostyle, corresponding to stigmatal rows 3-9. In Bsp18, purple pigment cells are concentrated around the dorsal edge of the dorsal lip. Purple pigment cells can also be found along the entire length of the endostyle. In Bsp17, magenta pigment cells are concentrated on the dorsal lip (either on the dorsal edge of the dorsal lip or across the entire dorsal lip), and around the oral siphon.

None of the zooids had ovaries, so we could not determine the position of the ovaries with respect to the testes. The left side testis is just anterior to the anterior-most intestinal loop, and the posterior portion of the right testis is overlapping with the cardiac ends of the anterior-most stomach folds. Therefore, the left side testis is anterior to the right side testis. In Bsp16, the left and right testis are directly across from one another. Number of lobes in testis is highly variable, between 10 and 26. When numerous, these lobes are not in a perfect rosette shape (e.g. lobes radiating out from a clearly defined center), but are clustered together in a sphere. Zooids have an average of 14.95 rows of stigmata on the left side, and an average of 14.88 rows of stigmata on the right side. The second stigmatal row is dorsally incomplete, on both the left and right side of the zooid. There are an average of 14.73 stigmata per half-row on the left side of the body, an average of 5.39 between the dorsal lamina and the first longitudinal vessel, 2.71 between the first and second longitudinal vessels, 2.59 between the second and third longitudinal vessels, and 4.03 between the third longitudinal vessel and the endostyle (DL 5.39 2.71 2.59 4.03 E). There are an average of 14.8 stigmata per half-row on the right side of the body, an average of 5.44 between the dorsal lamina and the first longitudinal vessel, 2.54 between the first and second longitudinal vessels, 2.51 between the second and third longitudinal vessels, and 4.3 between the third longitudinal vessel and the endostyle (DL 5.44 2.54 2.51 4.3 E).

The intestine is angular (base of the rectum and the intestine make an acute angle) (e.g. Plate 19b). The anterior edge of the intestinal loop extends to between the 10th stigmatal row and the 15th transverse vessel, with an average extension to between the 13th stigmatal row and the 13th transverse vessel. The anus opens between the 9th and 15th stigmatal rows, with an average opening at the 12th stigmatal row. The anus opens most commonly 1-1.5 stigmatal rows anterior to the anterior edge of the intestinal loop.

There are 9-10 stomach folds, with 10 being the most common number. The last stomach fold is sometimes smaller than the others. The stomach folds do not have longitudinal grooves. The stomach is conical, meaning that the pyloric end is narrower than the cardiac end (e.g. Plate 19c). The cardiac ends of the stomach folds have ovoid swellings (e.g. Plate 19c). The tip of the pyloric caecum bends slightly with respect to the base (e.g. Plate 16c). The tip of the pyloric caecum is moderately swollen (e.g. Plate 16c). The pyloric caecum is small (e.g. Plate 16c). There are 8 oral tentacles, with either 2 or 3 size orders. 3 size orders is most common, with a pattern such as L S M S L S M S.

There is an extensive and dense network of ampullae along the outer edge of the tunic and in the tunic below the zooids. In the colonies where the tunic is 5-6 mm thick, since the zooids are only 2 mm long there is a 3-4 mm thick section of the tunic at the base of the colony that does not contain zooids. The tunic is tough, not delicate. The tunic folds into lobes on the tops of the colonies, there are no zooids inside these lobes (e.g. Plate 18a, Plate 20a).

Supplementary Figure S2: Morphological characters assessed for botryllid ascidian species in this study. These characters are commonly used in the botryllid taxonomic literature, and were compiled from two articles: Brunetti 2009, Saito and Okuyama 2003.

1. Appearance of the colony: Circular systems or ladder-like systems

2. Position of the gonads: Ovary anterior/dorsal or posterior to testis.

3. Position of testes: Left testis anterior to, or directly across from, right testis.

4. Appearance of testes: Rosette-shaped or hemispherically-shaped

5. Number of testis lobes

6. Number of rows of stigmata (left side)

7. Number of rows of stigmata (right side)

8. Second stigmatal row: Dorsally complete or incomplete

9. Number of stigmata between Dorsal Lamina and 1st longitudinal vessel (Left Side)

10. Number of stigmata between 1st and 2nd longitudinal vessel (Left Side)

11. Number of stigmata between 2nd and 3rd longitudinal vessel (Left Side)

12. Number of stigmata between 3rd longitudinal vessel and endostyle (Left Side)

13. Number of stigmata between Dorsal Lamina and 1st longitudinal vessel (Right Side)

14. Number of stigmata between 1st and 2nd longitudinal vessel (Right Side)

15. Number of stigmata between 2nd and 3rd longitudinal vessel (Right Side)

16. Number of stigmata between 3rd longitudinal vessel and endostyle (Right Side)

17. Angle of the rectum with respect to the intestinal loop: ≤ 90 degrees, > 90 degrees

18. Anterior edge of intestinal loop extends to which stigmatal row

19. Anus opens at the level of which stigmatal row

20. Number of stigmatal rows between anterior edge of intestinal loop and anus

21. Number of stomach folds

22. Presence of longitudinal grooves in stomach folds

23. Shape of stomach: Conical or cylindrical

24. Presence of ovoid swellings on cardiac ends of stomach folds

25. Tip of caecum bent with respect to base of caecum

26. Size of pyloric caecum: Small (less than 1/4 the length of the stomach), medium (1/4-1/2 the length of the stomach) or large (more than 1/2 the length of the stomach)

27. Tip of pyloric caecum: Not swollen, slightly swollen, moderately swollen, swollen

28. Number of oral tentacles

29. Size orders of oral tentacles: 3 size orders (Small/Medium/Large), 2 size orders (Small/Large)

30. Zooid length (in mm)

31. Tunic thickness (in mm)

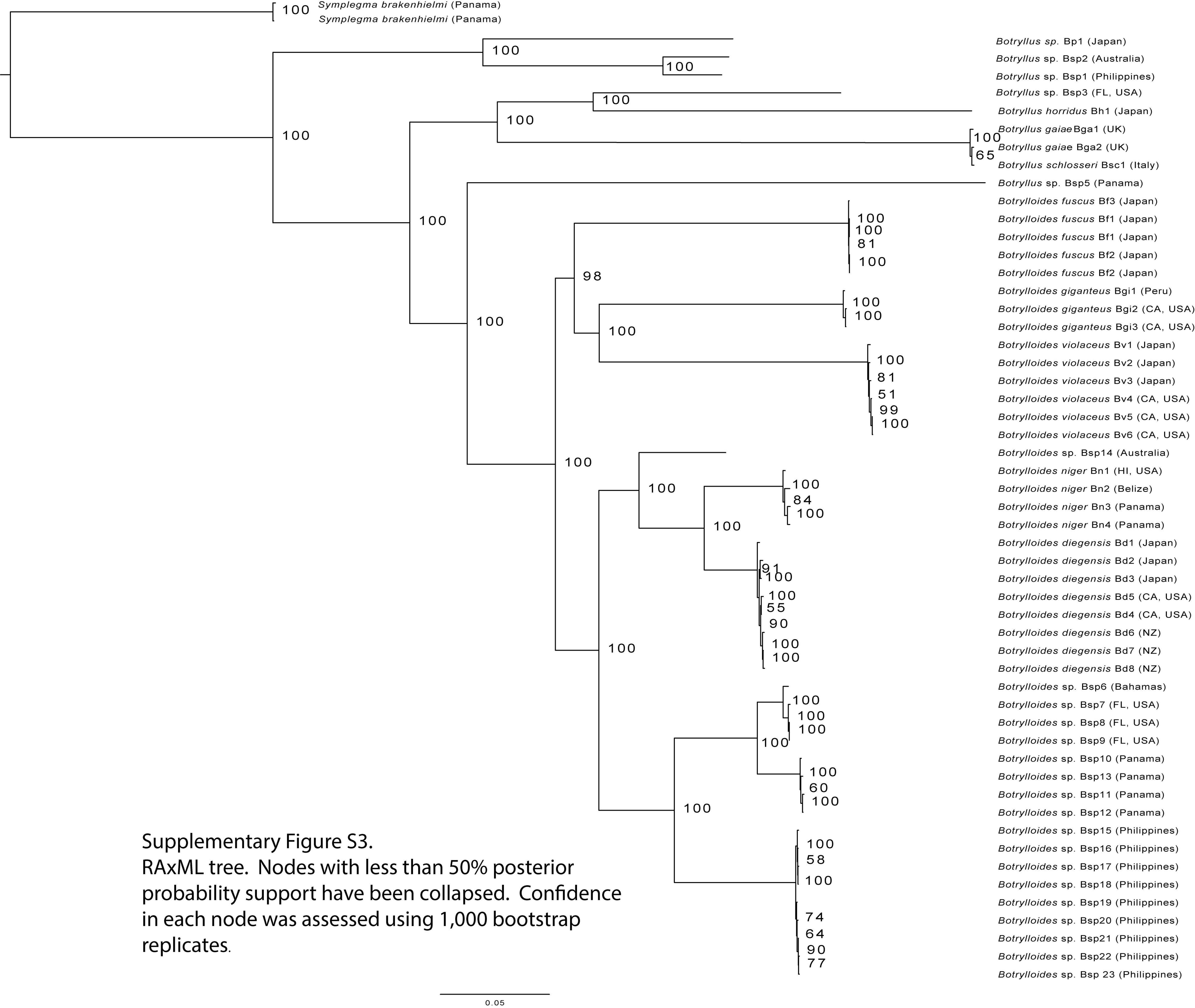

Supplementary Figure S3.
RAxML tree. Nodes with less than 50% posterior probability support have been collapsed. Confidence in each node was assessed using 1,000 bootstrap replicates.

Supplementary Figure S4: Photographs of each sample when available, including external colony morphology, zooids removed from tunic, and stomachs. The order of photographs in this figure follows Table 1. Unique specimen IDs from Table 1 are included in the Plate legends below.

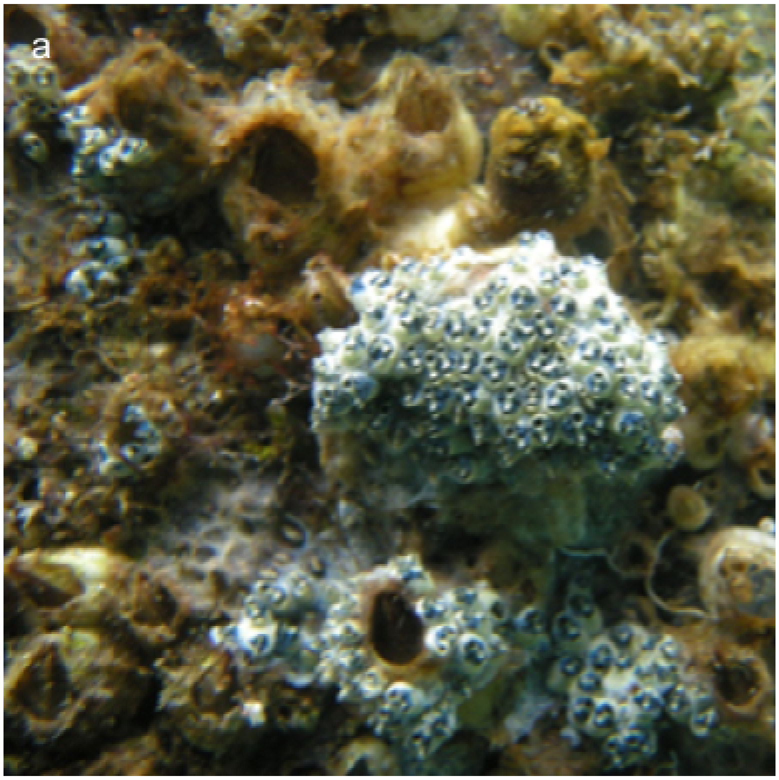

Plate 1: *Symplegma brakenhielmi* from Bocas del Toro, Panama, live colony (taken by Rosana Rocha).

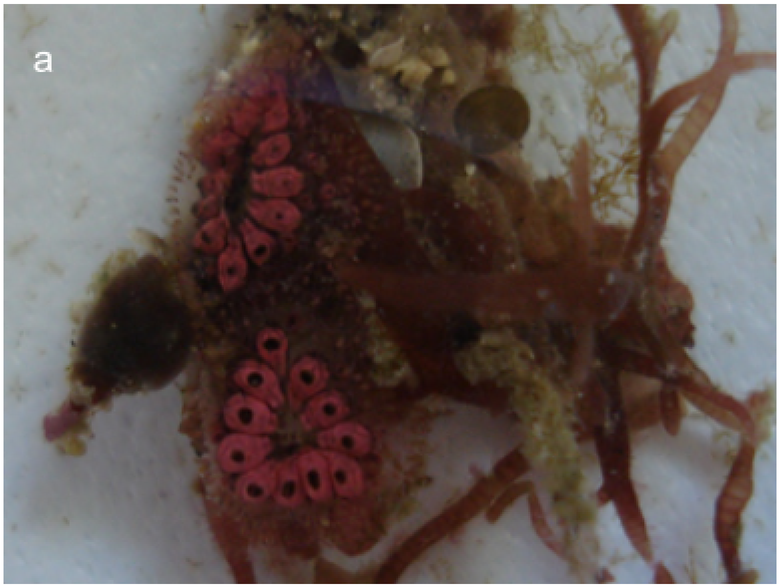

Plate 2: *Botryllus sp.* (Bsp1) from Maricaban Island, Philippines, live colony (taken by Beth Moore)

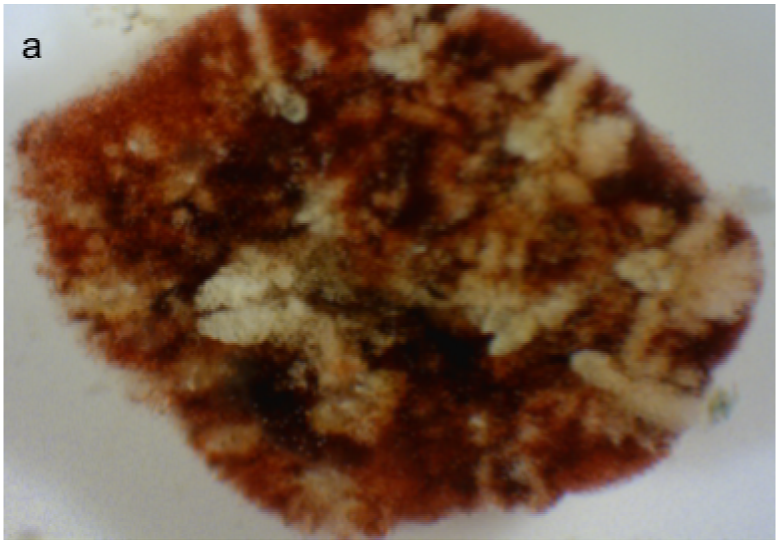

Plate 3: *Botryllus sp.* (Bsp2) from Heron Island, Australia, live colony (taken by Tony De Tomaso)

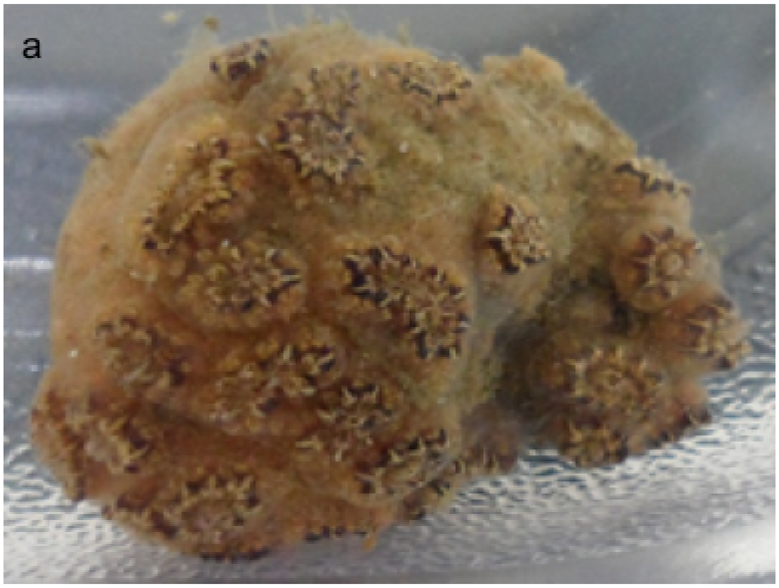

Plate 4: *Botryllus horridus* (Bh1) from Miura, Japan, live colony (taken by Marie Nydam)

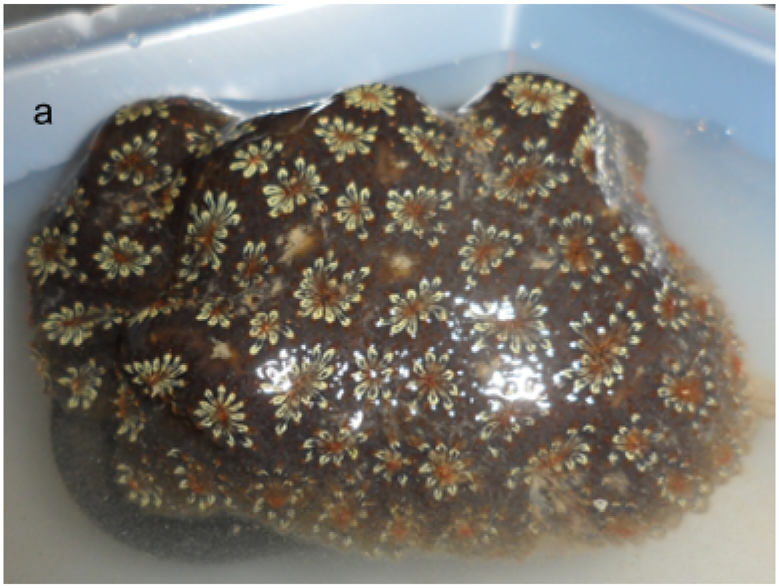

Plate 5: *Botryllus gaiae* from Granville, France, live colony (taken by Marie Nydam)

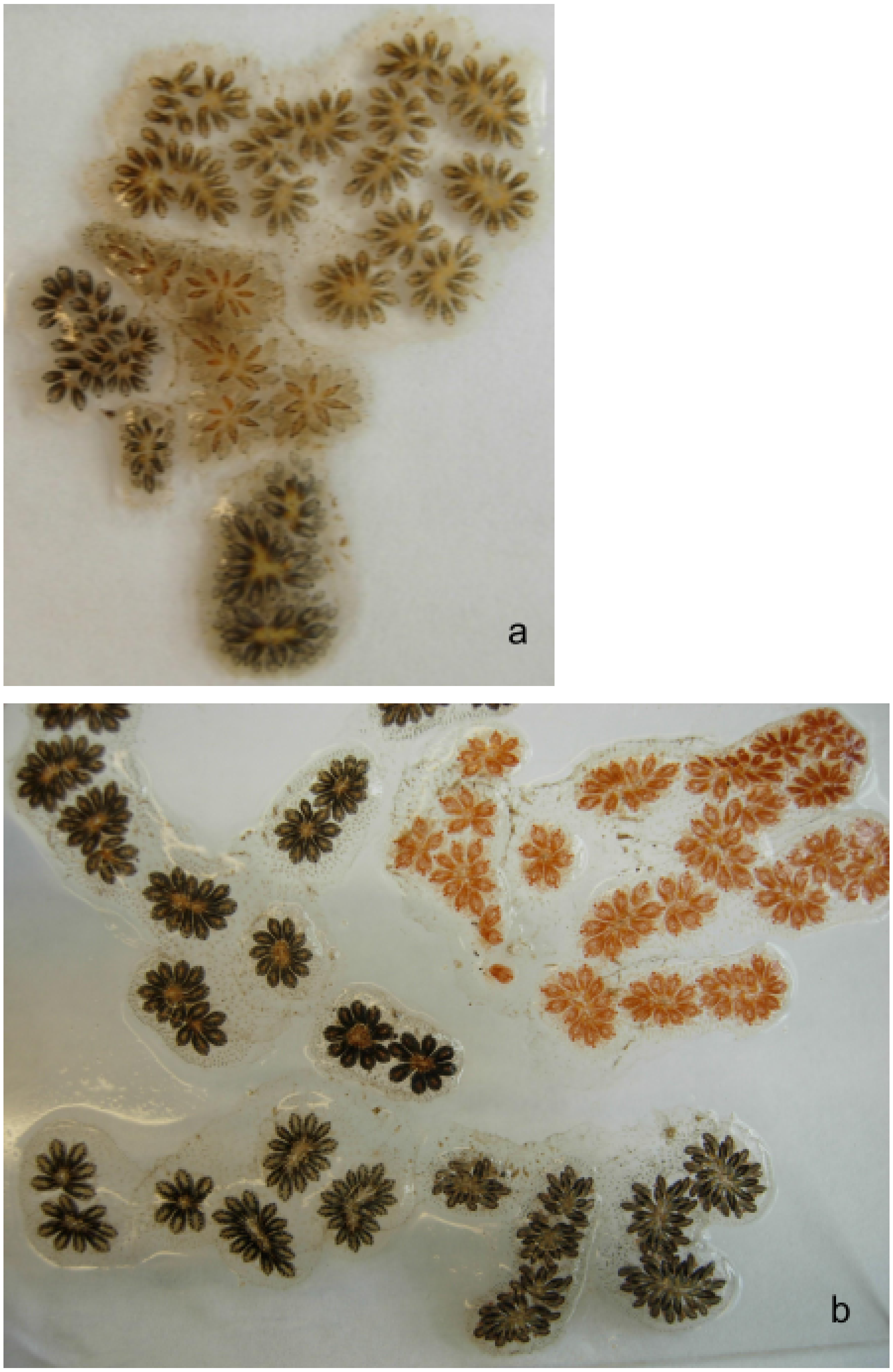

Plate 6: *Botryllus schlosseri* from Santa Barbara, CA, USA, live colonies (taken by Marie Nydam)

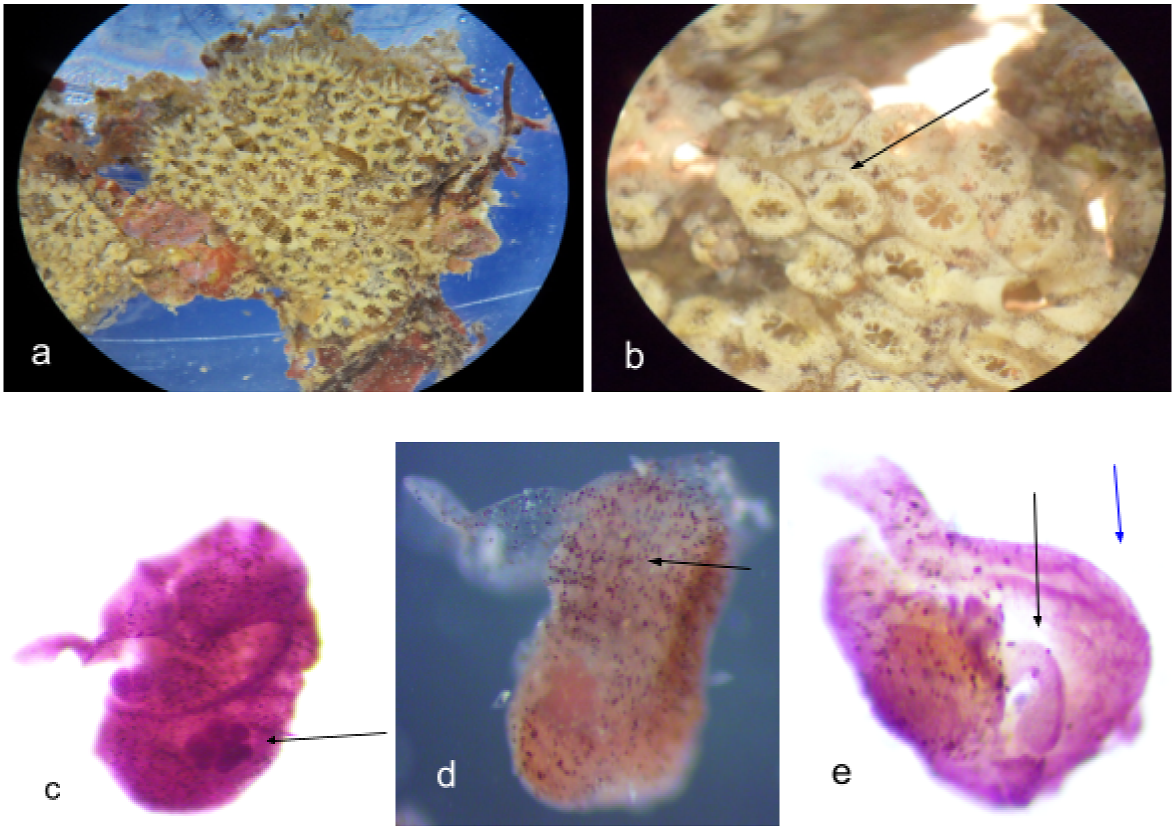

Plate 7: *Botryllus sp.* (Bsp5) from Bocas del Drago, Panama (taken by Marie Nydam). a) Live colony. b) Live colony. Arrow marks two black pigment clusters posterior to oral siphon. c) Stained zooid. Arrow marks right side testis in a rosette formation, and showing the posterior portion of the right testis is overlapping with the cardiac ends of the anterior-most stomach folds. d) Unstained zooid. Arrow marks purple pigment cells in transverse vessel. e) Stained stomach and intestine. Blue arrow shows angular intestine (the base of the rectum and the intestine make an acute angle). Black arrow shows pyloric caecum.

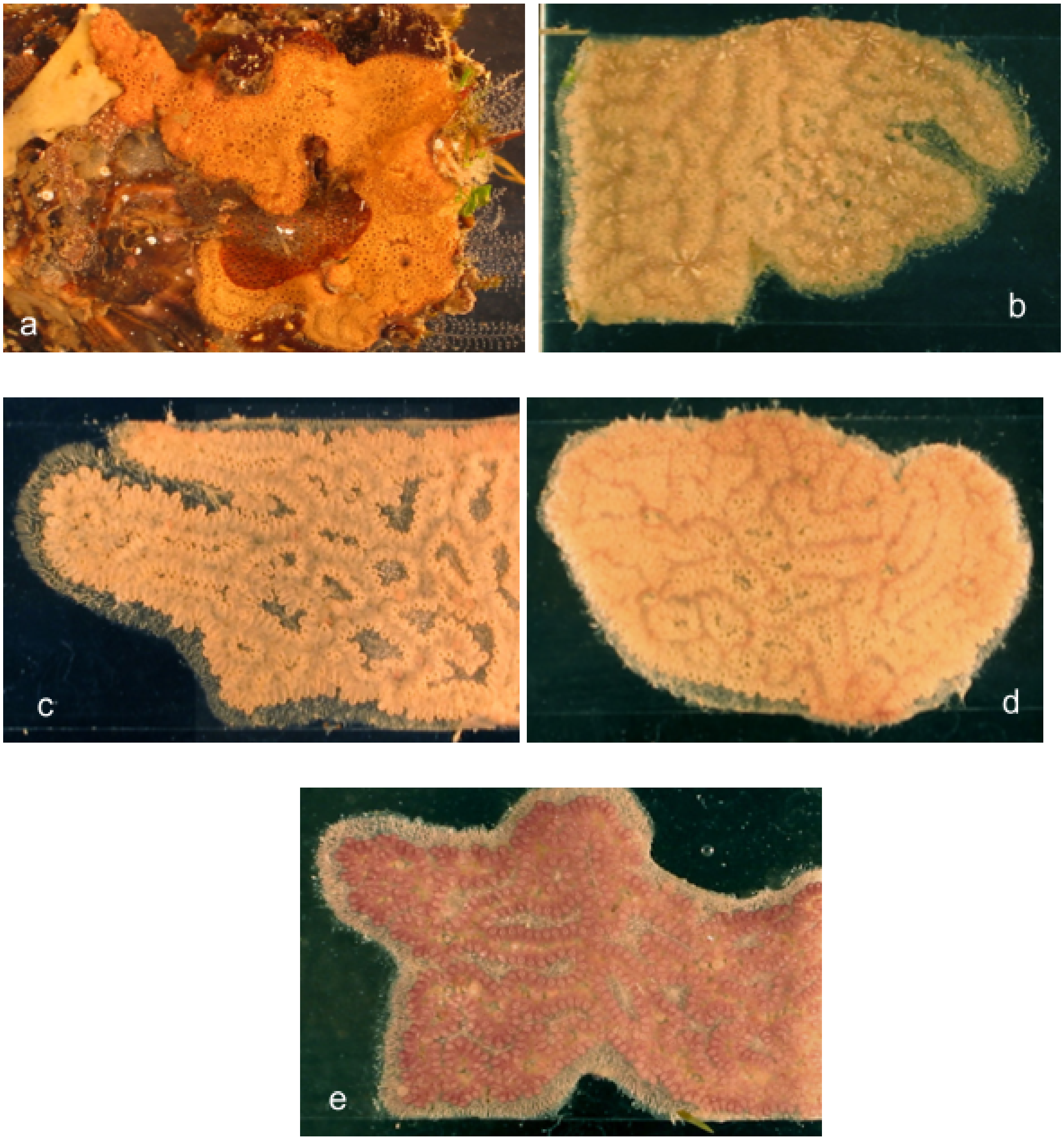

Plate 8: *Botrylloides violaceus* from Shimoda, Japan, live colonies (taken by Yas Saito).

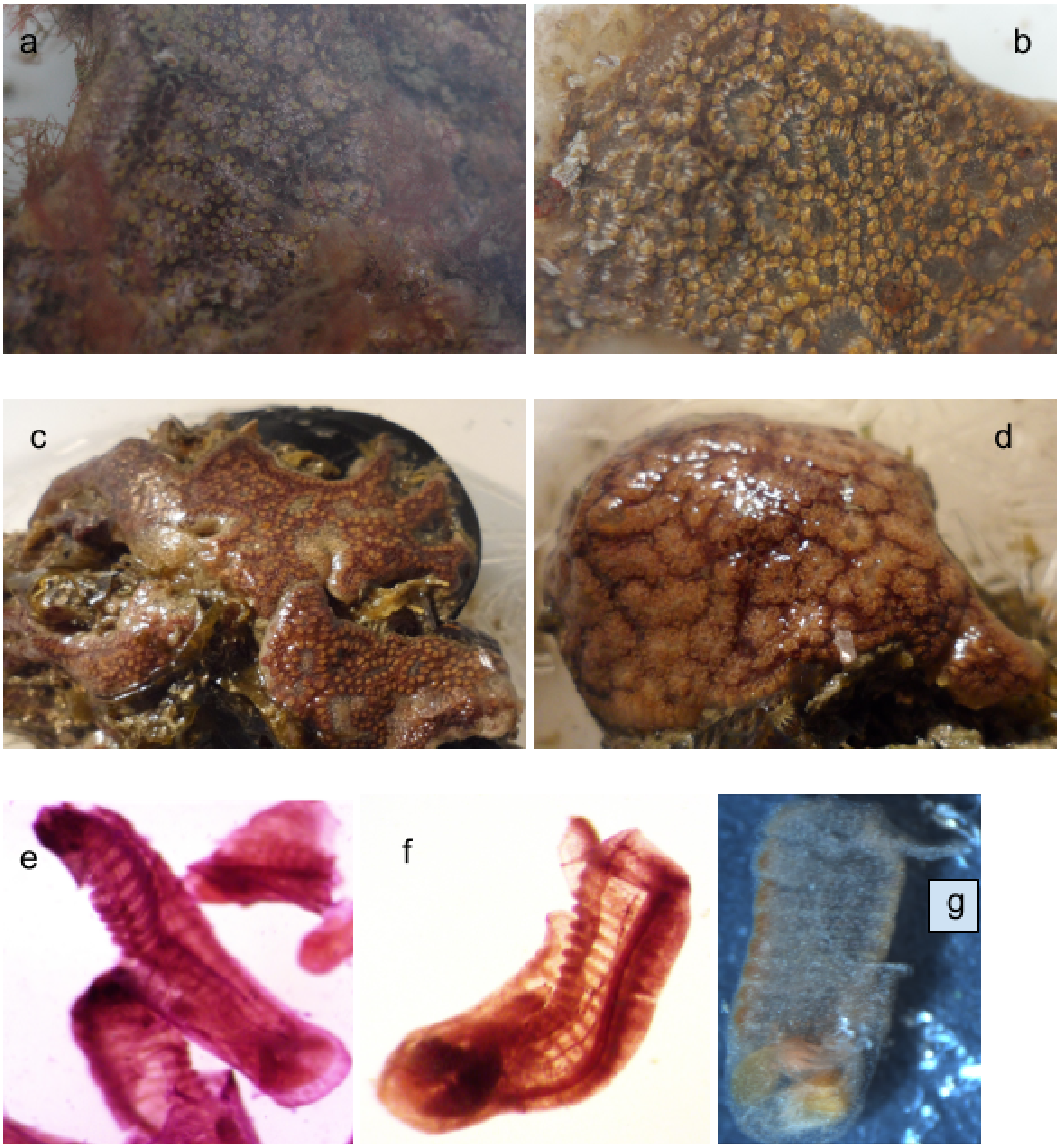

Plate 9: *Botrylloides giganteus*. a) Bgi2 from San Diego Bay, CA, USA, live colony (taken by Gail Ashton). b) Bgi3 from San Diego Bay, CA, USA, live colony (taken by Verena Wang). c) San Diego Bay, CA, USA, live colony (taken by Marie Nydam). d) San Diego Bay, CA, USA, live colony (taken by Marie Nydam). e) San Diego Bay, CA, USA, stained zooid (taken by Marie Nydam). f) San Diego Bay, CA, USA, unstained zooid (taken by Marie Nydam). g) San Diego Bay, CA, USA, unstained zooid (taken by Ritchelle Quiambao).

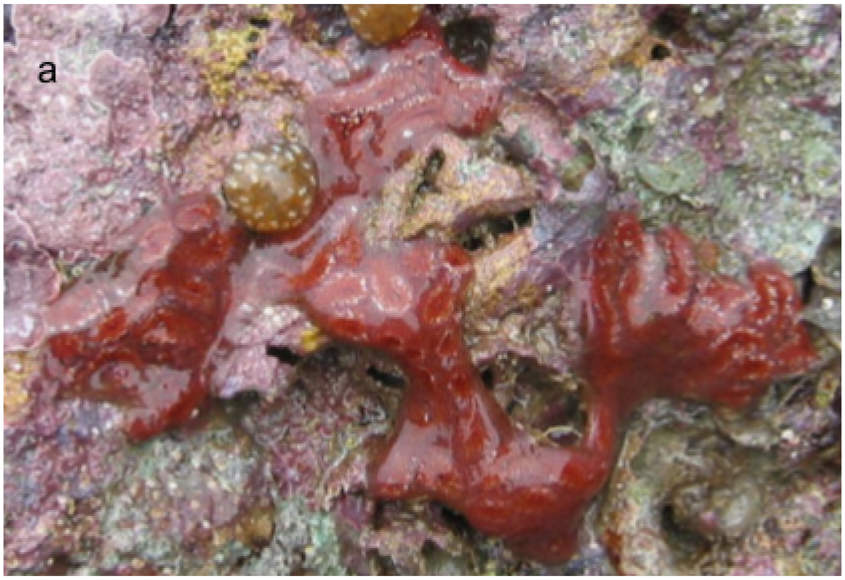

Plate 10: *Botrylloides fuscus* from Ebisu-jima, Japan, live colony (taken by Yas Saito)

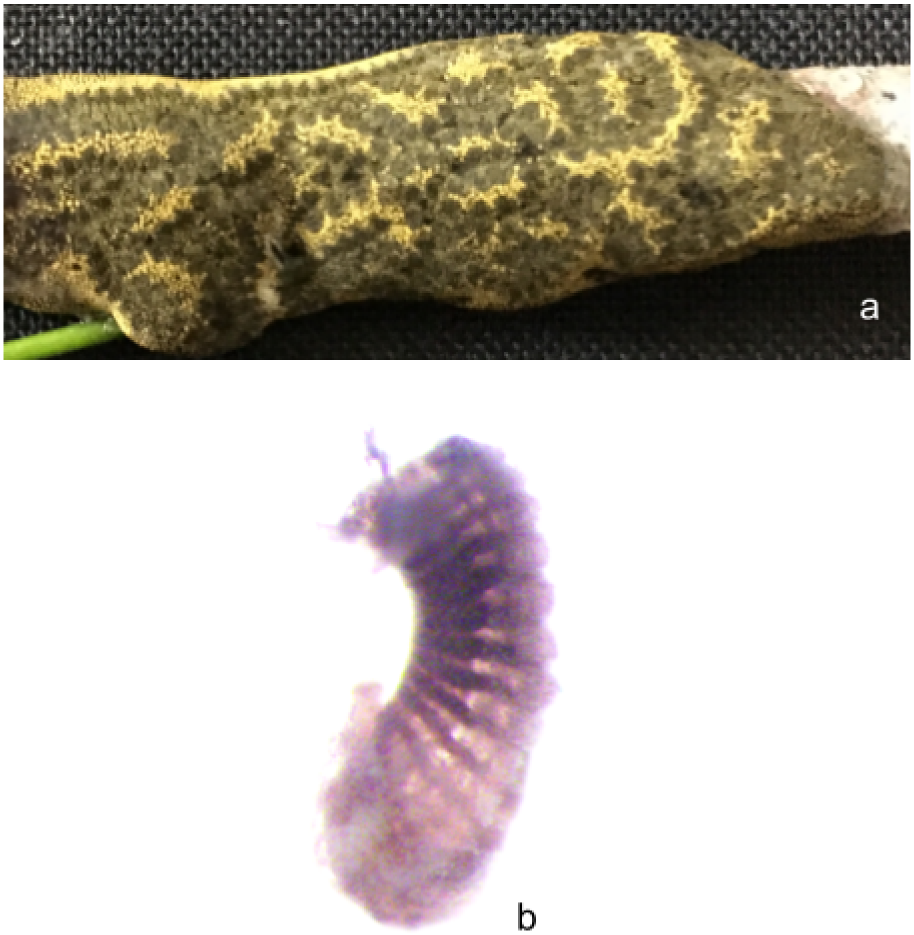

Plate 11: *Botrylloides sp.* Rabbit Key and Barnes Key, Florida, USA a) Live colony (taken by Tom Frankovich) b) Stained zooid (taken by Marie Nydam)

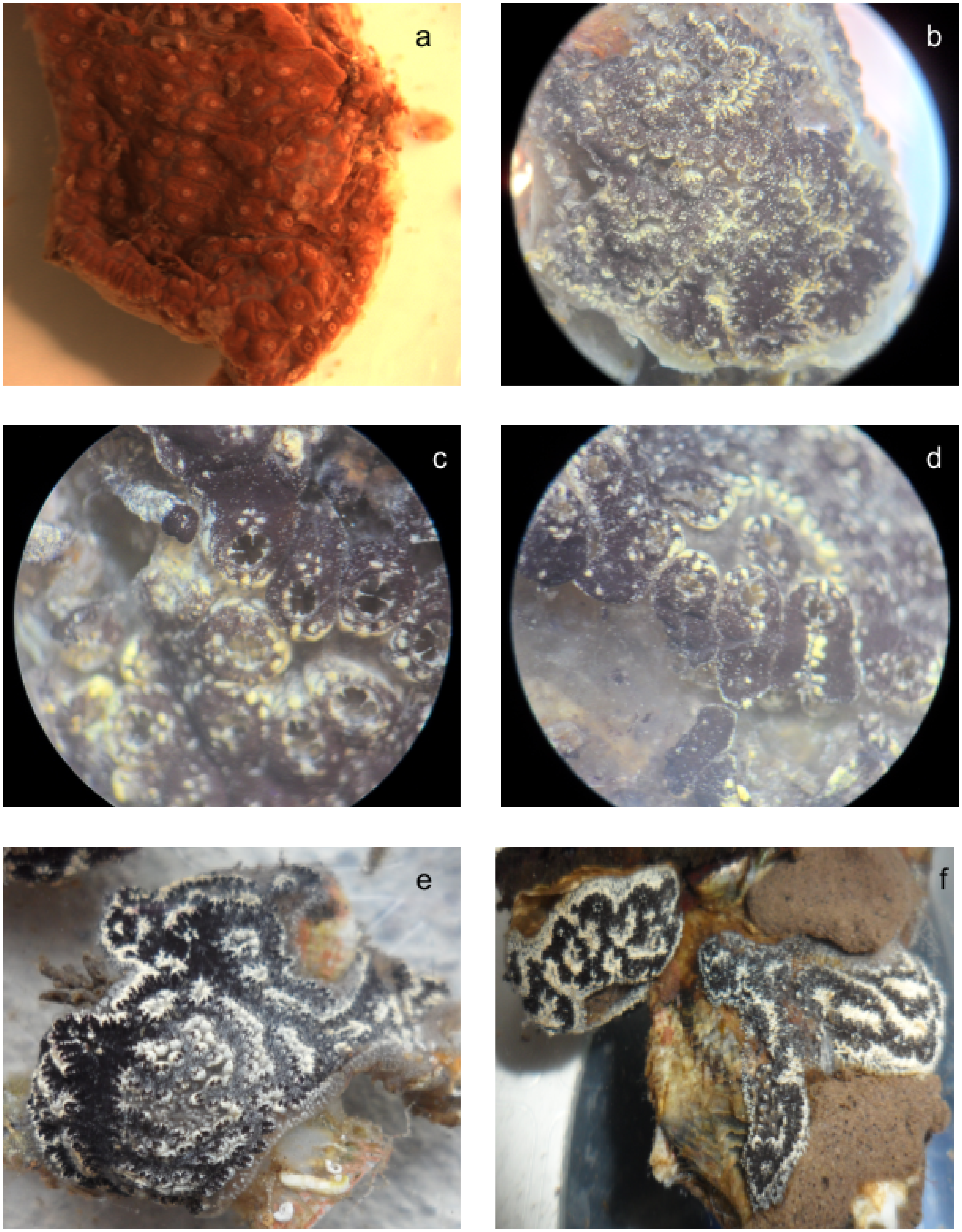
a
b
c
d
e
f

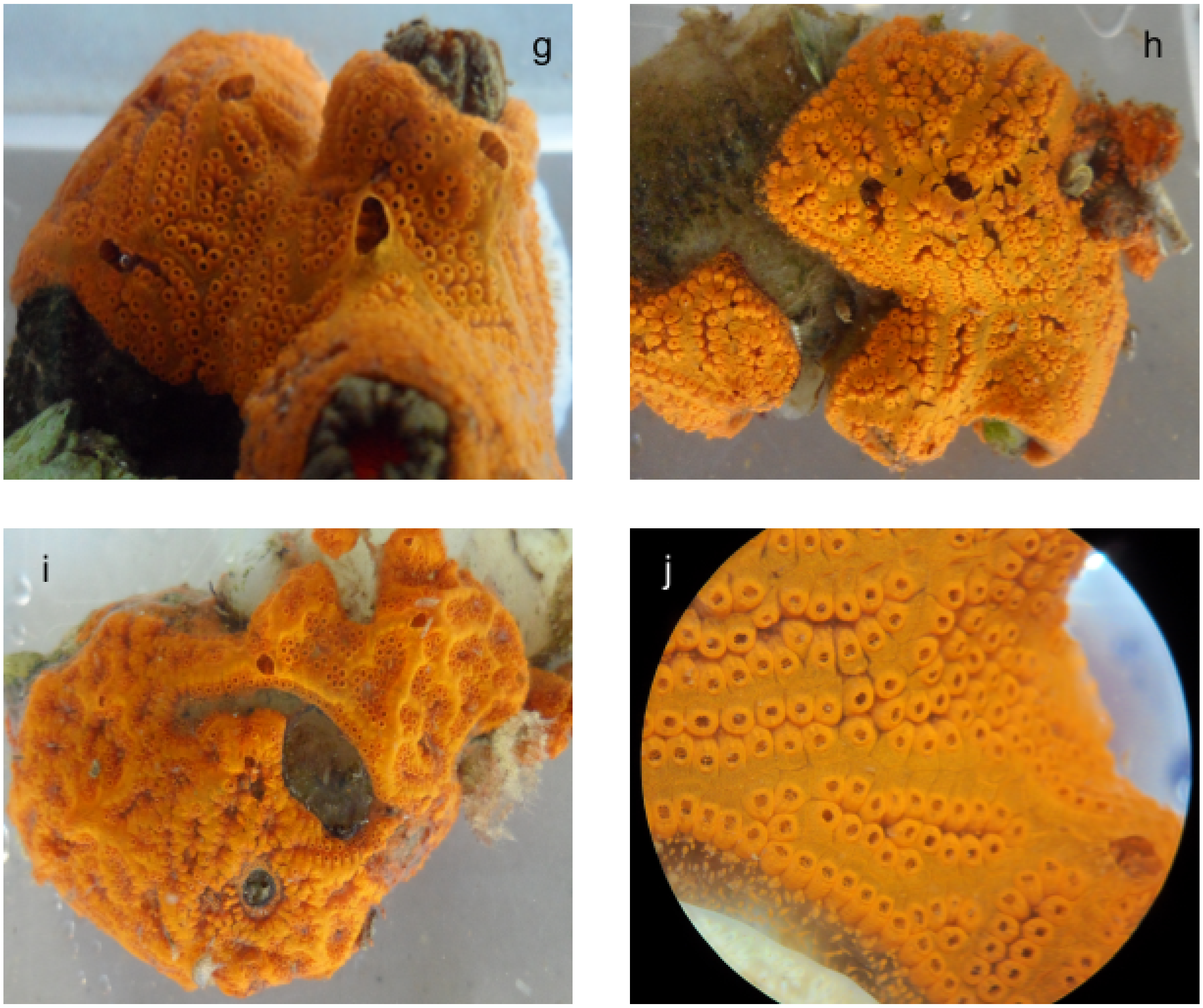
g
h
i
j

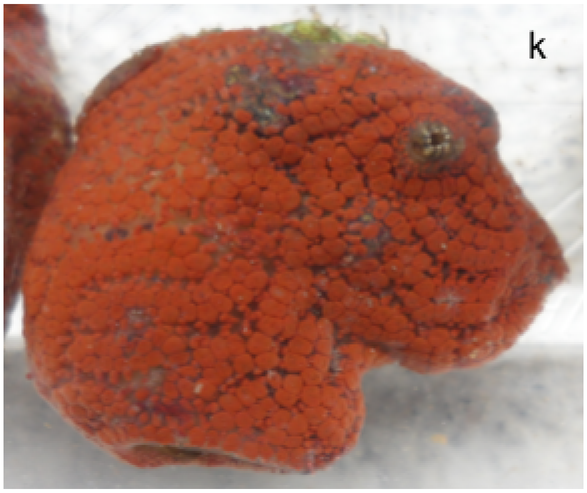
k

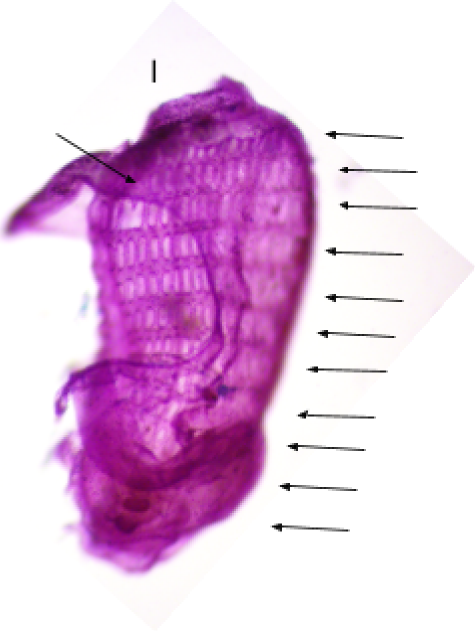
l

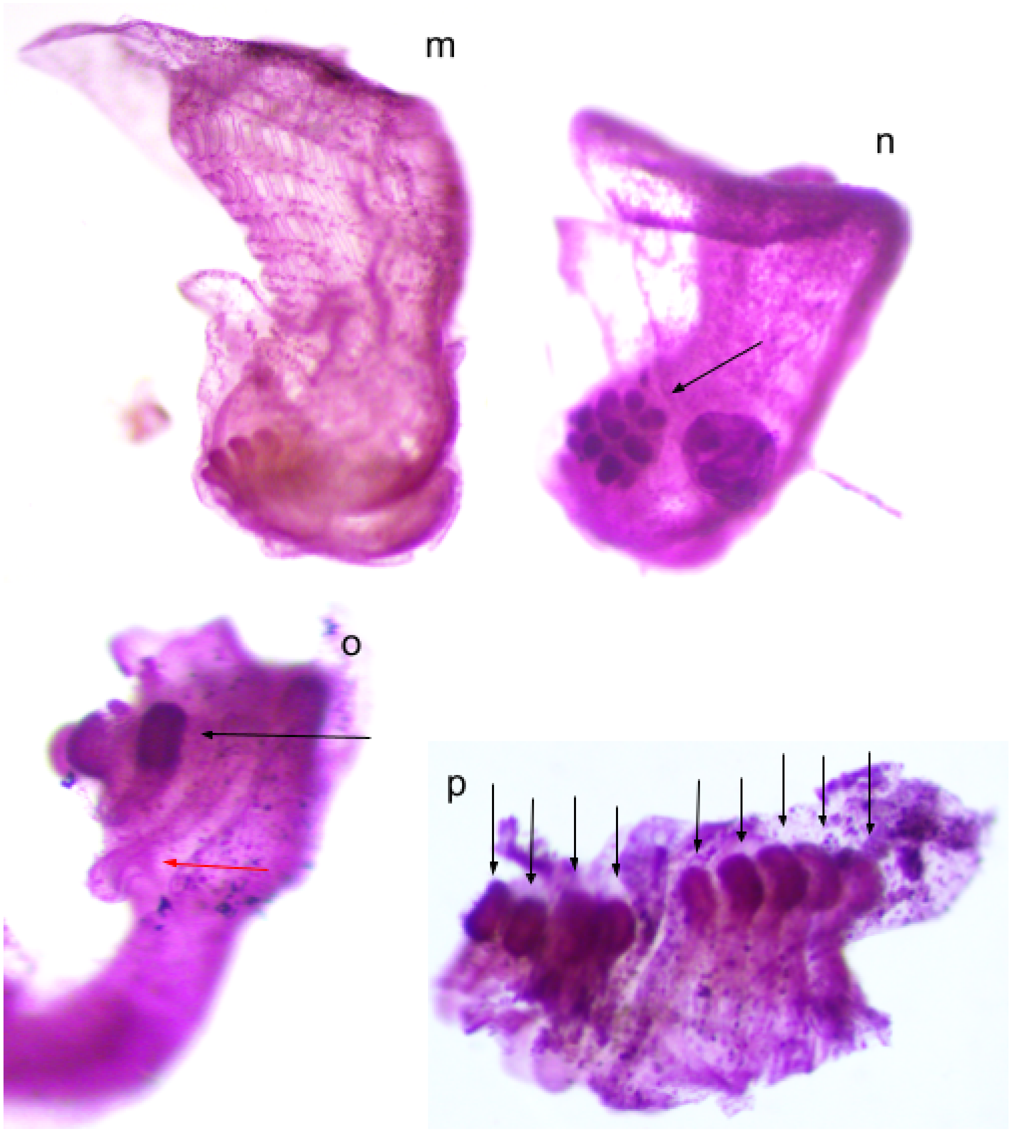

Plate 12: *Botrylloides sp.* from Panama. a) Bsp10 from Colon, Panama, preserved colony (taken by Greg Ruiz). b-e) Bsp12 from Bocas del Toro, Panama, live colony (taken by Marie Nydam). f) Bsp11 from Bocas del Toro, Panama, live colony (taken by Marie Nydam). g-j) From Bocas del Toro, Panama, live colony (taken by Marie Nydam). k) Bsp13 from Bocas del Toro, Panama, live colony (taken by Marie Nydam). l-m) From Bocas del Toro, Panama, stained zooid (taken by Marie Nydam). Arrows denote 11 stigmatal rows, and 2nd stigmatal row incomplete. n) Bsp13 from Bocas del Toro, Panama, stained zooid (taken by Marie Nydam). Arrows denote testis. o) Bsp13 from Bocas del Toro, Panama, stained stomach (taken by Marie Nydam). Red arrow marks the pyloric caecum, and black arrow the ovoid swellings of the stomach folds. p) From Bocas del Toro, Panama, stomach cut to show nine stomach folds (denoted with arrows).

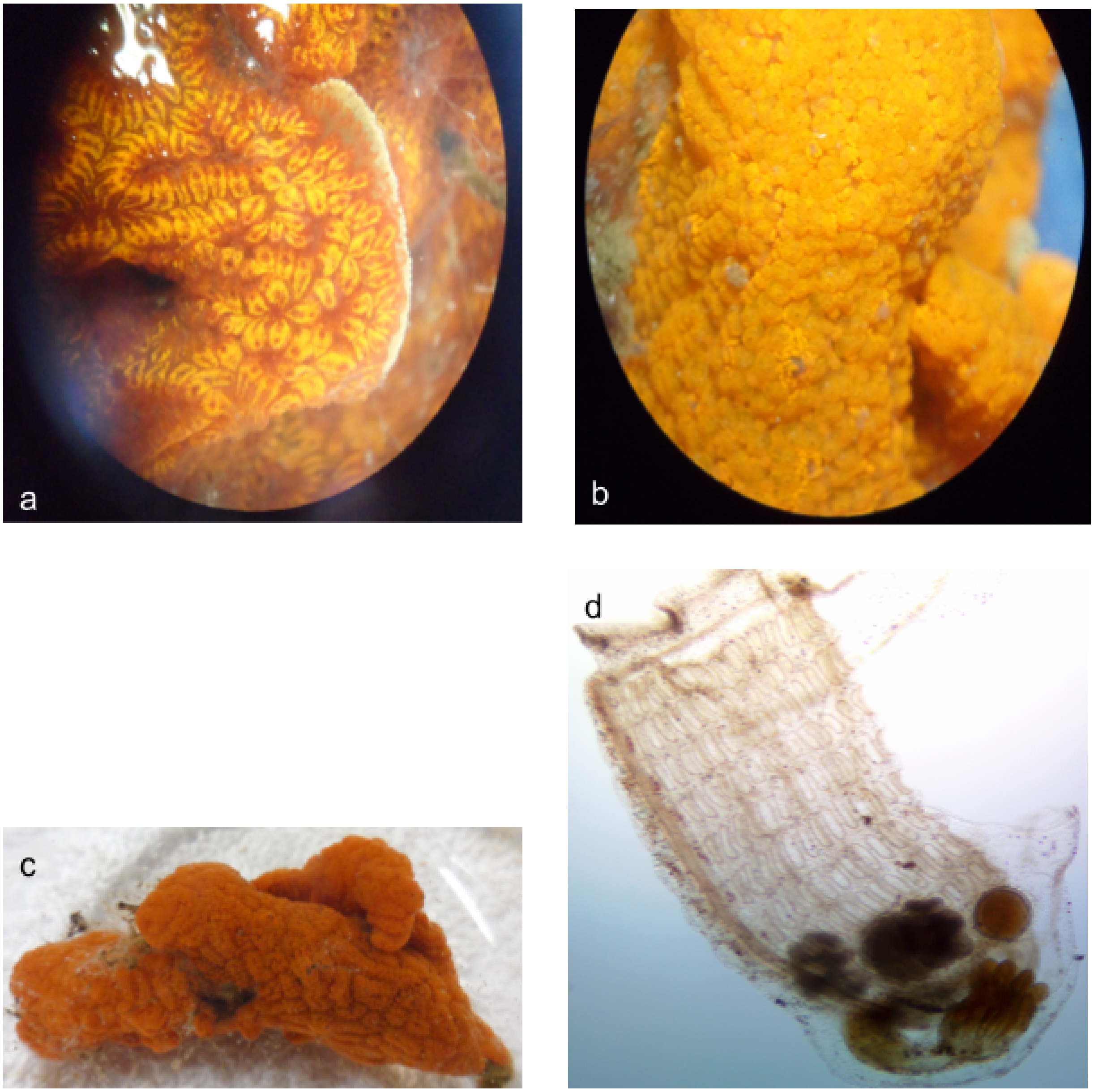

Plate 13: *Botrylloides niger* from Bocas del Toro, Panama. a) Bn4, live colony (taken by Marie Nydam). b-c) Bn3, live colony (taken by Marie Nydam). d) Bn3, unstained zooid (taken by Marie Nydam)

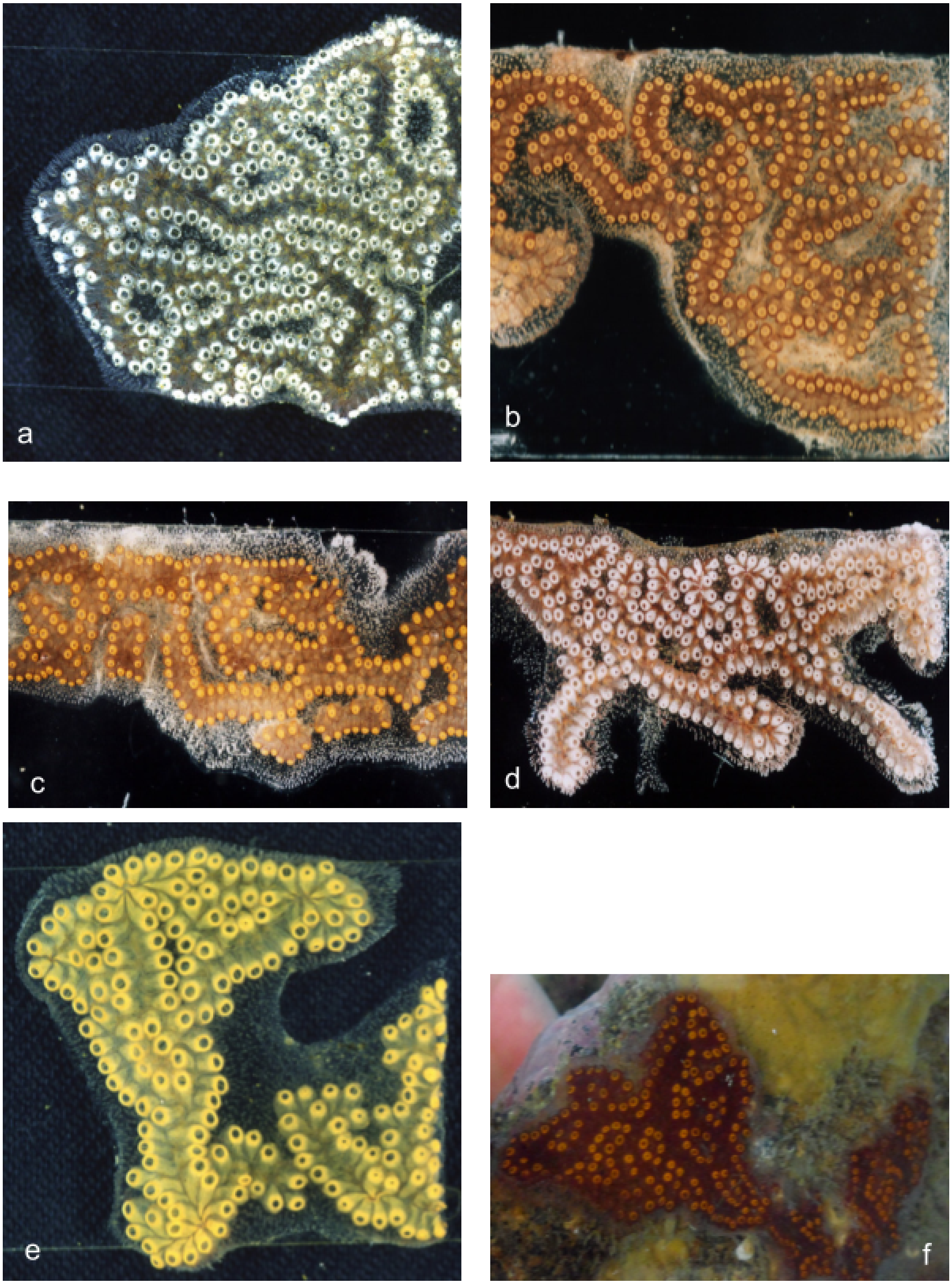
a
b
c
d
e
f

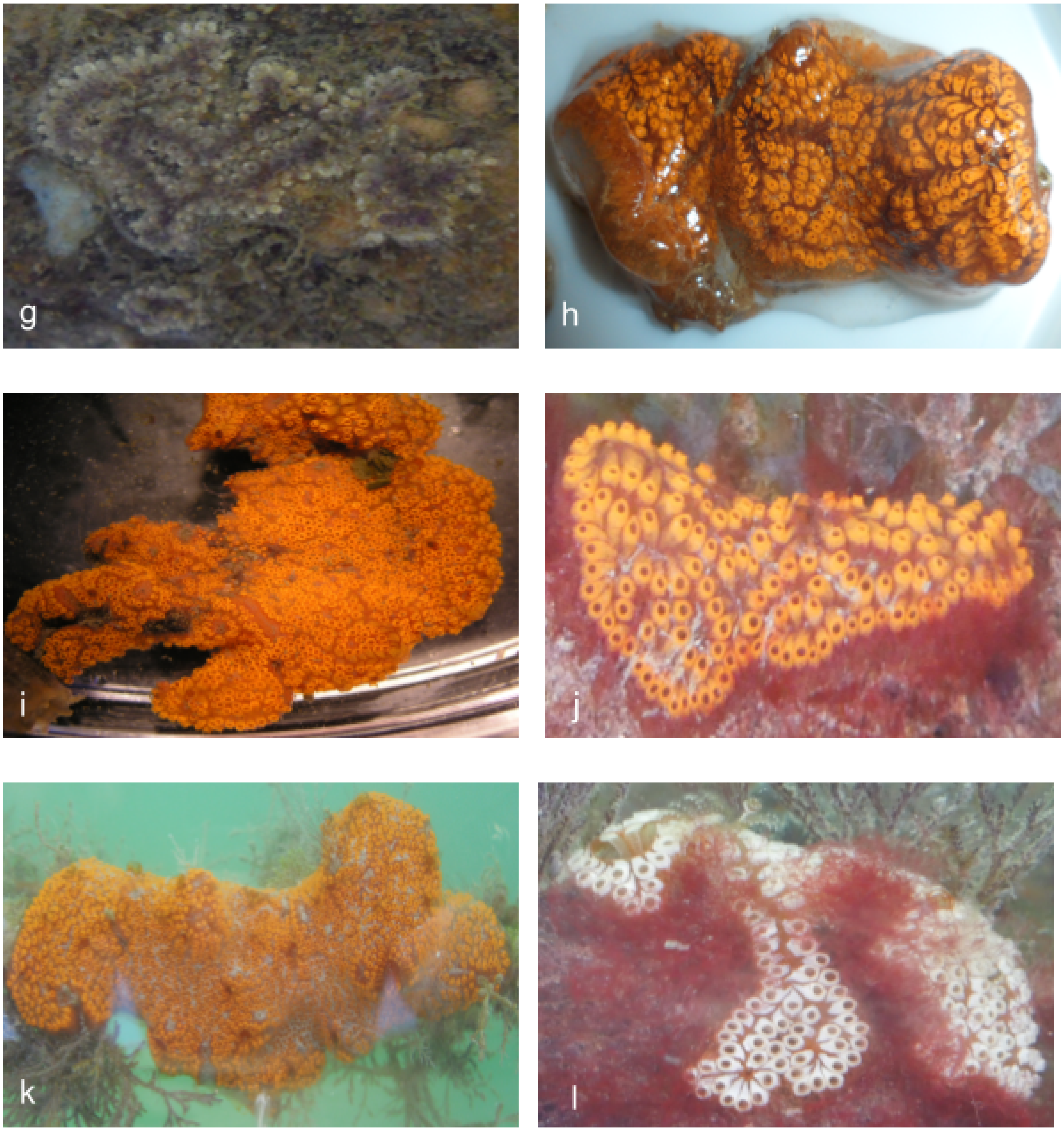

Plate 14: a-e) *Botrylloides diegensis* from Shimoda, Japan, live colonies (taken by Yas Saito). f-g) *Botrylloides diegensis* from Miura, Japan, live colonies (taken by Marie Nydam). h) *Botrylloides diegensis* from Granville, France, live colony (taken by Marie Nydam). i) *Botrylloides diegensis* from Bodega Bay, CA, USA, live colony (taken by Jay Stachowicz). j-l) *Botrylloides diegensis* from Port Nelson, New Zealand, live colonies (taken by Gretchen Lambert).

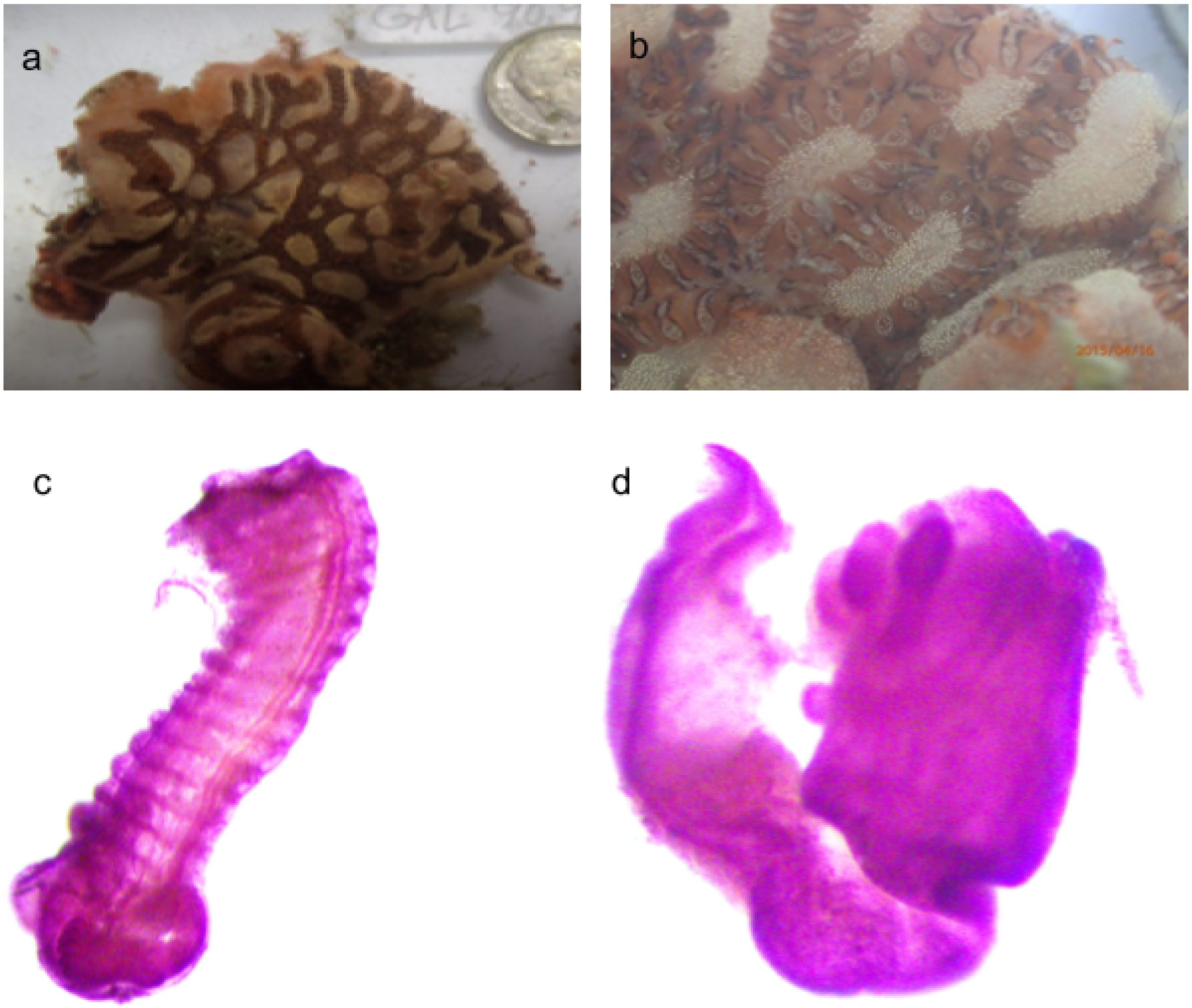

Plate 15: *Botrylloides sp.* (Bsp15) from Medio Island, Philippines. a-b) Live colonies (taken by Sarah Cohen). c) Stained zooid (taken by Marie Nydam). d) Stained stomach (taken by Marie Nydam)

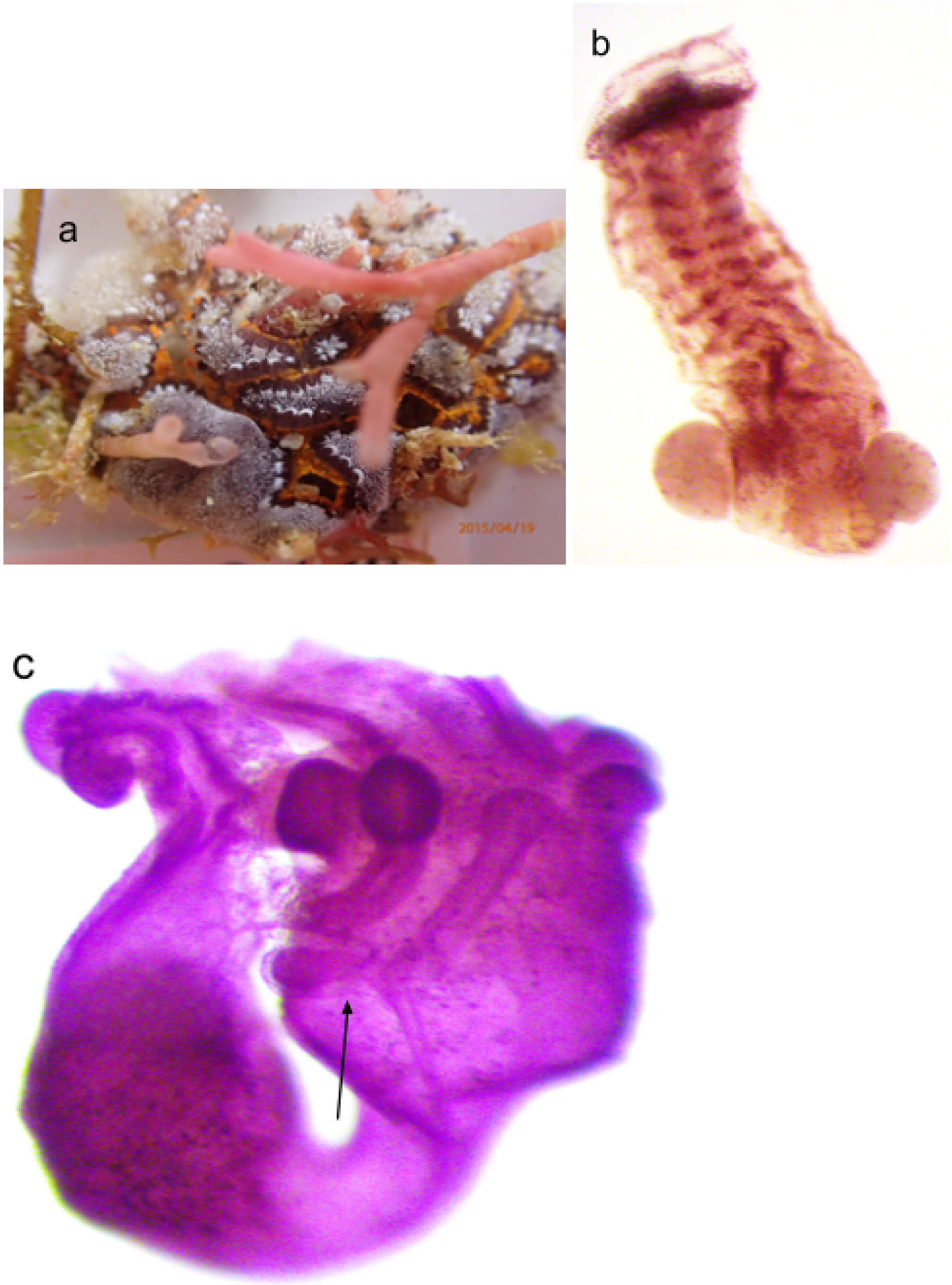

Plate 16: *Botrylloides sp.* (Bsp16) from Manila Channel, Puerto Galera, Philippines. a) Live colony (taken by Sarah Cohen). b) Unstained zooid (taken by Marie Nydam). c) Stained stomach (taken by Marie Nydam). Arrow marks pyloric caecum.

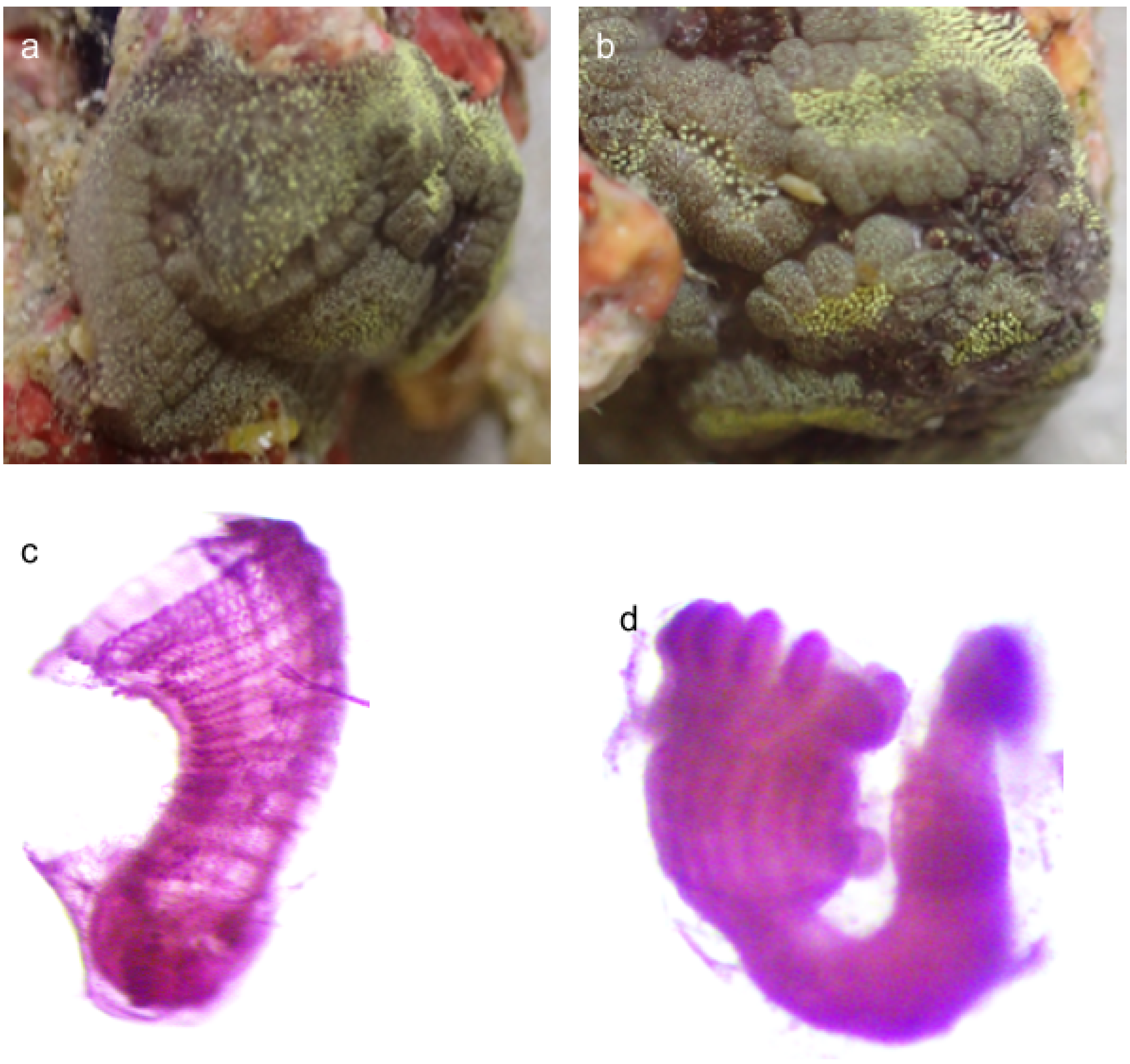

Plate 17: *Botrylloides sp.* (Bsp17) from Batangas Channel, Puerto Galera, Philippines. a-b) Live colony (taken by Sarah Cohen). c) Stained zooid (taken by Marie Nydam). d) Stained stomach (taken by Marie Nydam).

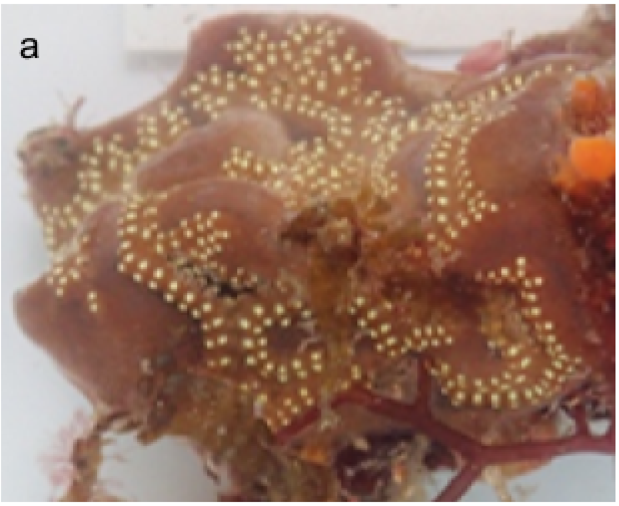

Plate 18: *Botrylloides sp.* (Bsp19) from Maricaban Island, Philippines, live colony (taken by Sarah Cohen)

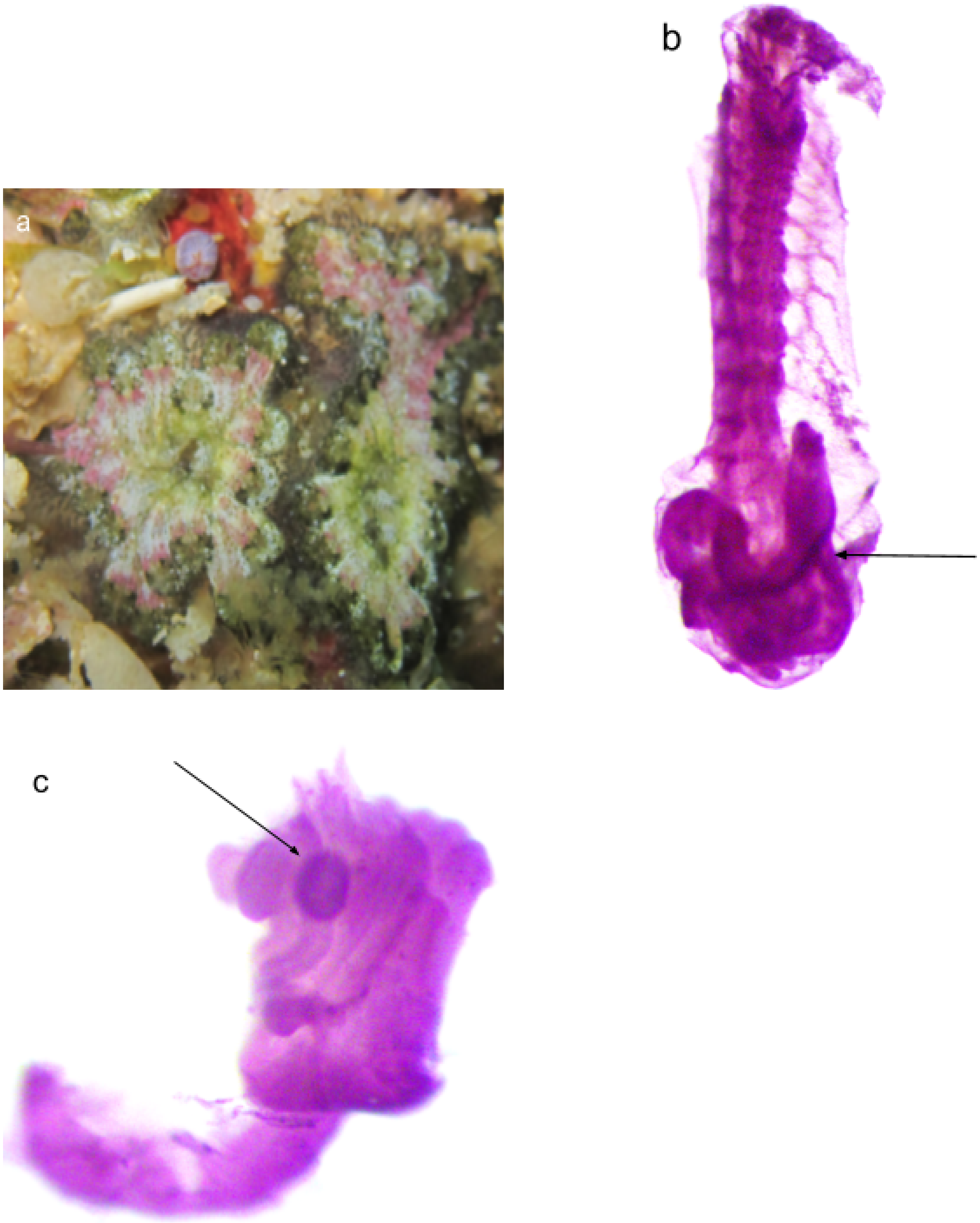

Plate 19: *Botrylloides sp.* (Bsp21) from Maricaban Island, Philippines. a) Live colony (taken by Chrissy Piotrowski). b) Stained zooid (taken by Marie Nydam). Arrow marks angular intestinal loop (the base of the rectum and the intestine make an acute angle). c) Stained stomach (taken by Marie Nydam). Arrow marks ovoid swelling at cardiac end of stomach fold.

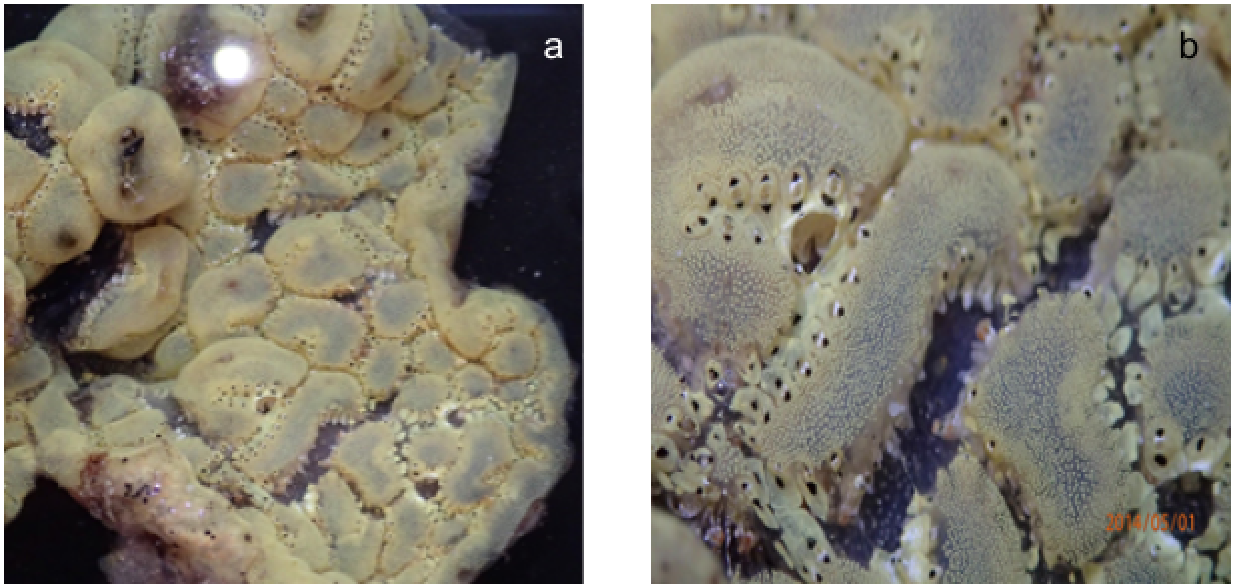

Plate 20: *Botrylloides sp.* (Bsp22) from Maricaban Island, Philippines, live colony (taken by Sarah Cohen)

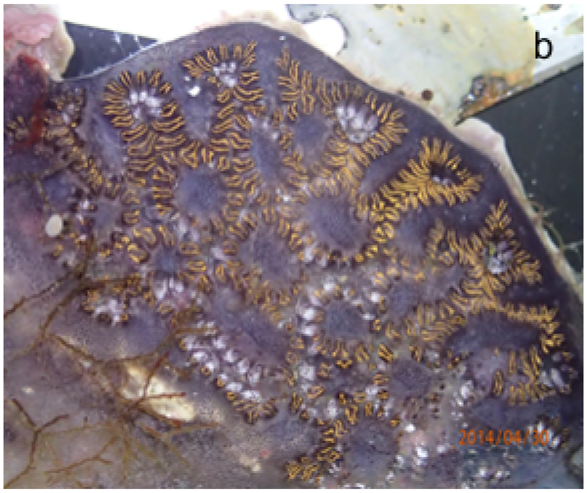

Plate 21: *Botrylloides sp.* (Bsp23) from Maricaban Island, Philippines, live colony (taken by Sarah Cohen)

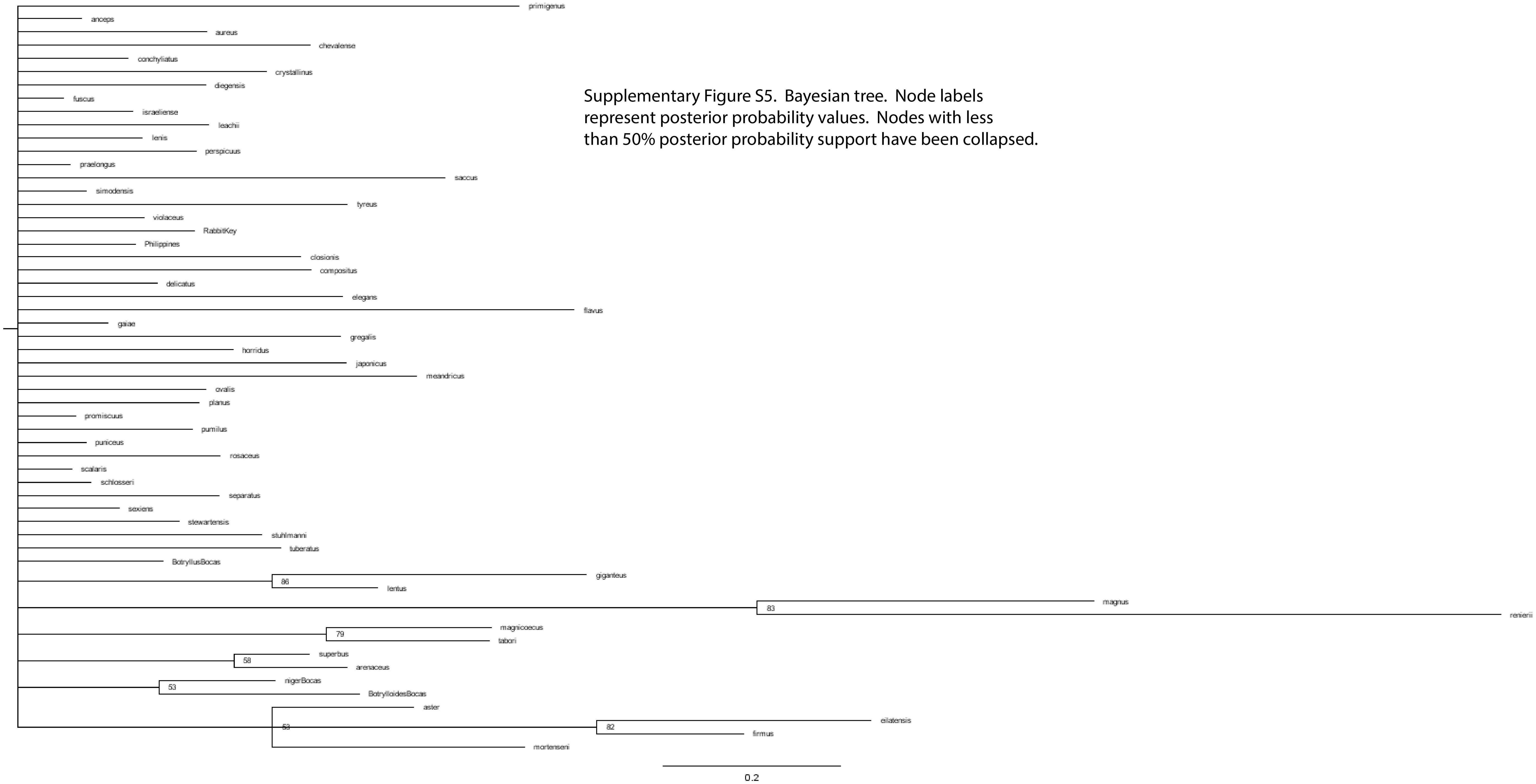

Supplementary Figure S5. Bayesian tree. Node labels represent posterior probability values. Nodes with less than 50% posterior probability support have been collapsed.
